# Identification and experimental validation of KMO as a critical immune-associated mitochondrial gene in unstable atherosclerotic plaque

**DOI:** 10.1186/s12967-024-05464-5

**Published:** 2024-07-18

**Authors:** Fu-Jun Liao, Shao-Liang Shen, Hai-Long Bao, Hui Li, Quan-Wei Zhao, Long Chen, Cai-Wei Gong, Cheng-Zhu Xiong, Wu-Peng Liu, Wei Li, Da-Nan Liu

**Affiliations:** 1 Department of Cardiovascular Medicine, The Affiliated Hospital of Guizhou Medical University, No. 28 Guiyi Street, Yunyan District, Guiyang, Guizhou, 550004 China; 2 Institute of Medical Sciences, Guizhou Medical University, No. 28 Guiyi Street, Yunyan District, Guiyang, Guizhou 550004 China; 3 School of Graduate Studies, Guizhou Medical University, No. 28 Guiyi Street, Yunyan District, Guiyang, Guizhou 550004 China; 4 The Key Laboratory of Myocardial Remodeling Research, The Affiliated Hospital of Guizhou Medical University, No. 28 Guiyi Street, Yunyan District, Guiyang, Guizhou, 550004 China

**Keywords:** Weighted Gene Co-expression network analysis, Unstable atherosclerotic plaques, Immune cell subtype distribution pattern, Kynurenine 3-monooxygenas, Hub genes

## Abstract

**Background:**

The heightened risk of cardiovascular and cerebrovascular events is associated with the increased instability of atherosclerotic plaques. However, the lack of effective diagnostic biomarkers has impeded the assessment of plaque instability currently. This study was aimed to investigate and identify hub genes associated with unstable plaques through the integration of various bioinformatics tools, providing novel insights into the detection and treatment of this condition.

**Methods:**

Weighted Gene Co-expression Network Analysis (WGCNA) combined with two machine learning methods were used to identify hub genes strongly associated with plaque instability. The cell-type identification by estimating relative subsets of RNA transcripts (CIBERSORT) method was utilized to assess immune cell infiltration patterns in atherosclerosis patients. Additionally, Gene Set Variation Analysis (GSVA) was conducted to investigate the potential biological functions, pathways, and mechanisms of hub genes associated with unstable plaques. To further validate the diagnostic efficiency and expression of the hub genes, immunohistochemistry (IHC), quantitative real-time polymerase chain reaction (RT-qPCR), and enzyme-linked immunosorbent assay (ELISA) were performed on collected human carotid plaque and blood samples. Immunofluorescence co-staining was also utilized to confirm the association between hub genes and immune cells, as well as their colocalization with mitochondria.

**Results:**

The CIBERSORT analysis demonstrated a significant decrease in the infiltration of CD8 T cells and an obvious increase in the infiltration of M0 macrophages in patients with atherosclerosis. Subsequently, two highly relevant modules (blue and green) strongly associated with atherosclerotic plaque instability were identified. Through intersection with mitochondria-related genes, 50 crucial genes were identified. Further analysis employing least absolute shrinkage and selection operator (LASSO) logistic regression and support vector machine recursive feature elimination (SVM-RFE) algorithms revealed six hub genes significantly associated with plaque instability. Among them, *NT5DC3*, *ACADL*, *SLC25A4*, *ALDH1B1*, and *MAOB* exhibited positive correlations with CD8 T cells and negative correlations with M0 macrophages, while kynurenine 3-monooxygenas (*KMO*) demonstrated a positive correlation with M0 macrophages and a negative correlation with CD8 T cells. IHC and RT-qPCR analyses of human carotid plaque samples, as well as ELISA analyses of blood samples, revealed significant upregulation of *KMO* and *MAOB* expression, along with decreased *ALDH1B1* expression, in both stable and unstable samples compared to the control samples. However, among the three key genes mentioned above, only *KMO* showed a significant increase in expression in unstable plaque samples compared to stable plaque samples. Furthermore, the expression patterns of *KMO* in human carotid unstable plaque tissues and cultured mouse macrophage cell lines were assessed using immunofluorescence co-staining techniques. Finally, lentivirus-mediated *KMO* silencing was successfully transduced into the aortas of high-fat-fed ApoE-/- mice, with results indicating that *KMO* silencing attenuated plaque formation and promoted plaque stability in ApoE-/- mice.

**Conclusions:**

The results suggest that *KMO*, a mitochondria-targeted gene associated with macrophage cells, holds promise as a valuable diagnostic biomarker for assessing the instability of atherosclerotic plaques.

**Supplementary Information:**

The online version contains supplementary material available at 10.1186/s12967-024-05464-5.

## Introduction

Atherosclerosis, a chronic inflammatory disease, is characterized by the progressive formation of plaques within arterial walls [1]. These plaques primarily comprise cholesterol, lipids, calcium, and various cellular components, gradually accumulating on the vessel wall. As these plaques grow in size over time, they can cause vascular stenosis and obstruction [2]. Acute coronary syndrome (ACS), transient ischemic attack, and ischemic stroke (IS) are acute cardiovascular events strongly linked to unstable atherosclerotic plaques, often triggered by plaque rupture. The rupture of an unstable plaque poses a life-threatening risk, leading to myocardial infarction (MI) and IS. Key characteristics of unstable plaques include a thin fibrous cap, a large necrotic core, infiltration of inflammatory cells, neovascularization within the plaque, intraplaque hemorrhage, and the presence of calcified nodules [3]. Early detection and identification of unstable plaques are crucial due to their elevated risk of rupture.

Mitochondria, characterized by a double membrane structure, are semi-autonomous organelles widely acknowledged as the cellular “powerhouses” crucial for maintaining cellular homeostasis. The impairment of mitochondrial function, marked by escalated reactive oxygen species generation, mitochondrial oxidative stress, compromised mitochondrial dynamics, and energy supply, has increasingly been linked to the onset and progression of atherosclerosis [4]. Research has highlighted the role of mitochondrial oxidative metabolism in the selective activation of macrophages [5]. The intricate involvement of immune cells, including monocytes, macrophages, T cells, and B cells, in atherosclerosis is critical, considering that they are recruited to sites of lipid accumulation. The interplay between immune cells and lipids initiates a cascade of inflammatory responses, culminating in the formation of atherosclerotic plaques [6, 7]. Studies have specifically identified CD4 + T cells as active participants in atherosclerosis, engaging in interactions with antigen-presenting cells like dendritic cells and macrophages [8]. Using cell-type identification by estimating relative subsets of RNA transcripts (CIBERSORT), a widely employed bioinformatics tool, facilitates the exploration of immune cell infiltration patterns and enables the assessment of the proportions of 22 distinct immune cell types using RNA-seq or microarray data [9]. However, limited research has been conducted on the infiltration patterns of immune cells and the identification of immune-related mitochondrial genes within carotid plaques of atherosclerosis patients. Therefore, evaluating immune cell infiltration patterns within carotid plaques of atherosclerosis patients shows promise for elucidating the intricate molecular mechanisms governing immunoregulation in atherosclerosis.

Weighted Gene Co-expression Network Analysis (WGCNA) is a robust systems biology informatics approach used to examine network relationships, decipher molecular mechanisms, and uncover potential key genes implicated in disease progression [10, 11]. Additionally, it facilitates exploration of the interactions between gene modules and sample characteristics, thereby identifying module genes significantly associated with features of interest in samples. Currently, the Search Tool for the Retrieval of Interacting Genes (STRING) online database and cytoHubba software are frequently employed to identify hub genes among several module genes significantly associated with features of interest in samples [12]. In a previous study led by Zheng et al., several key genes significantly associated with coronary heart disease were successfully identified through the integration of WGCNA with protein-protein interaction and cytoHubba analysis methods [13]. However, the selection process of key genes, such as choosing the top-ranked genes by degree value [12, 13] or the top 5 or 10 differentially expressed genes [14, 15], depends on the researcher’s preference, which may reduce the accuracy and reproducibility of the selection process. To address this issue, various machine learning techniques have recently been incorporated into bioinformatics analyses, demonstrating improved accuracy and stability in the selection method [16]. The least absolute shrinkage and selection operator (LASSO) regression, as a normalized linear regression method, allows for the exclusion of unimportant features and the construction of sparse and interpretable models to prevent overfitting. The support vector machine-recursive feature elimination (SVM-RFE) technique integrates support vector machines into a recursive feature elimination strategy, leveraging the inherent feature selection capability of support vector machines to select key features during iterative iterations. LASSO regression and SVM-RFE algorithms, widely recognized machine learning techniques, are frequently utilized for the identification of key genes. The combination of LASSO and SVM-RFE techniques has demonstrated satisfactory accuracy and sensitivity in certain fields, such as lung tumors and pituitary tumors [17]. However, limited studies have employed the combined application of WGCNA, LASSO, and SVM-RFE to identify hub genes linked to unstable atherosclerotic plaques.

To accomplish our goals, this study first performed WGCNA to identify key module genes associated with unstable atherosclerotic plaques. Subsequently, mitochondrial genes were selected from these identified key modules for further analysis. A combination of LASSO regression and SVM-RFE was then employed to identify hub mitochondria-related genes associated with unstable atherosclerotic plaques. The biological functions of these key mitochondria-related genes were evaluated using Gene Set Variation Analysis (GSVA). The infiltration patterns of immune cells in carotid plaque samples obtained from patients diagnosed with unstable atherosclerotic plaques were examined using the CIBERSORT method. Additionally, the relationship between multiple hub genes and 22 types of immune cells was investigated. Furthermore, validation of the expression patterns, diagnostic efficacy, cellular localization, and biological functions of key mitochondrial genes was conducted using techniques such as immunohistochemistry (IHC), Enzyme-Linked Immunosorbent Assay (ELISA), immunofluorescence co-staining, and lentiviral transfection, based on human carotid plaque samples, blood samples, and the Apolipoprotein E knockout (ApoE-/-) mice model of atherosclerosis.

## Materials and methods

### Data collection and preprocessing of atherosclerosis datasets

**Fig. 1:**
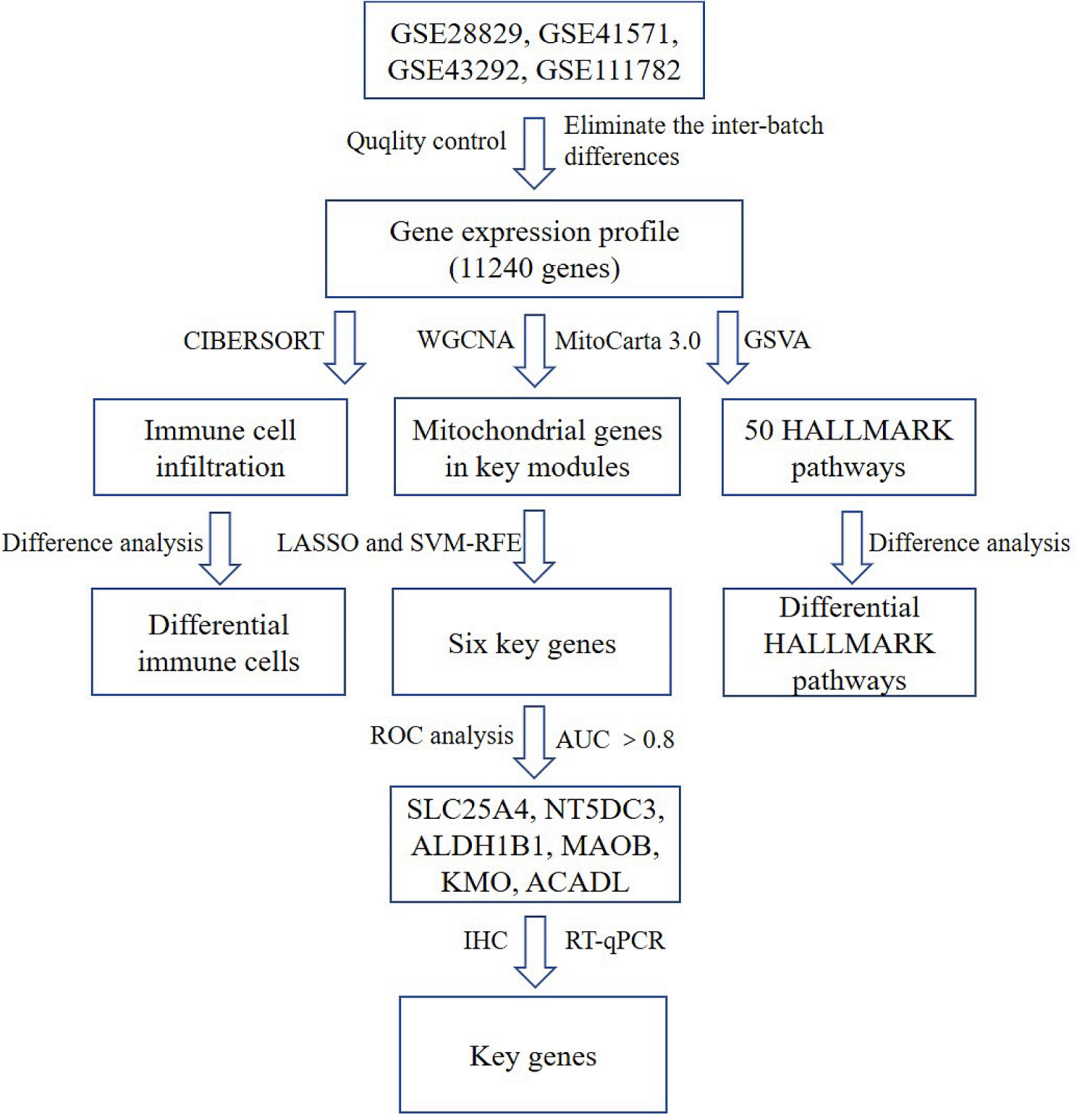
Flowchart of the analysis. WGCNA, Weighted gene co-expression network analysis; LASSO, Least absolute shrinkage and selection operator; SVM-RFE, Support vector machine-recursive feature elimination; GSVA, Gene Set Variation Analysis; ROC, Receiver Operating Characteristic; IHC, immunohistochemistry; RT-qPCR, Quantitative real time polymerase chain reaction; AUC, areas under the curve; *NT5DC3*, 5’-nucleotidase domain containing 3; *ACADL*, acyl-CoA dehydrogenase long chain; *SLC25A4*, solute carrier family 25 member4; *ALDH1B1*, aldehyde dehydrogenase 1 family member B1; *MAOB*, mono amineoxidase B; *KMO*, kynurenine3-monooxygenase

Gene expression profiles were retrieved from the publicly accessible Gene Expression Omnibus (GEO) database at http://www.ncbi.nlm.nih.gov/geo using “atherosclerosis” as the primary keyword. Specific datasets related to atherosclerosis expression patterns, namely GSE28829, GSE41571, GSE43292, and GSE111782, along with platform data such as GPL570, GPL571, and GPL6244, were obtained. Additionally, a set of mitochondrial genes was extracted from the MitoCarta 3.0 database [18]. The GSE28829 dataset included expression data from 13 cases of early-stage and 16 cases of late-stage atherosclerotic plaques. GSE41571 comprised expression data from 6 stable plaques and 5 unstable plaques. GSE43292 contained expression data from 32 macroscopically intact arterial tissues and 32 plaque tissues. Lastly, GSE111782 encompassed expression data from 9 asymptomatic and 9 symptomatic patients’ plaques. To ensure consistency and eliminate batch effects between the datasets, the integrated gene expression profiles were standardized. This involved employing the “normalize Between Arrays” function from the limma package to normalize the gene expression data. Moreover, the “ComBat” function from the “sva” R package was utilized to remove any remaining batch effects between the datasets [19]. The entire workflow is visually depicted in Fig. 1.

### Identification of modules associated with unstable atherosclerotic plaques through WGCNA

The standardized and batch-corrected integrated gene expression profiles underwent WGCNA to explore the top 25% of genes displaying substantial expression differences. A scale-free network was constructed to determine the appropriate soft-thresholding power (soft threshold = 14). Next, a power function was utilized to compute the connection values between genes exhibiting variances surpassing the lower quartile of all variances. Subsequently, the connection values were transformed into a topological overlap matrix (TOM), from which dissimilarity values (1-TOM) were derived. The dynamic tree-cutting method was then applied for hierarchical clustering of genes, utilizing 1-TOM as the distance metric, a depth split value of 2, and a minimum module size cut-off of 100, to identify distinct modules [19]. A threshold of 0.25 was applied to merge modules exhibiting high similarity. Pearson’s correlation tests were conducted to evaluate the relationship between clinical characteristics and modules, aiming to identify significant modules. Module membership (MM) was defined as the correlation between the gene expression profile and module eigengenes. Gene significance (GS) was defined as the absolute value of the correlation between external traits and gene expression profiles. Subsequently, modules significantly associated with advanced or ruptured plaques, and exhibiting a significant correlation between MM and GS values (with an absolute correlation coefficient greater than 0.3), were defined as meaningful modules for further analysis [12, 19].

### Machine learning

To identify potential key genes, the overlap between module genes associated with ruptured plaques in atherosclerosis and genes related to mitochondria was obtained. Two distinct machine learning algorithms, namely LASSO and SVM-RFE, were then employed to identify hub genes possessing optimal prognostic value for unstable atherosclerotic plaques. LASSO logistic regression analysis was performed using the “glmnet” package, with a binomial response type and alpha set to 1. Moreover, the SVM-RFE algorithm was utilized to iteratively eliminate feature vectors generated by the support vector machine, employing threshold settings of halve.above = 100 and k = 5, to identify the most informative variables [19]. Lastly, the intersection of genes obtained from both algorithms was determined to identify the hub genes associated with unstable atherosclerotic plaques.

### Immune infiltration analysis

The present study adopted the “Cibersort” algorithm to transform the standardized gene expression matrix, enabling the characterization of infiltrating immune cell composition to investigate immune infiltration patterns in atherosclerosis [20]. Visualization tools, such as the “ggplot2” package, were used to generate correlation heatmaps and violin plots, effectively illustrating disparities in immune cell infiltration between the control group and atherosclerosis patients. Additionally, the “corrplot” package was applied to compute Pearson correlation coefficients among immune cell populations and visualize the outcomes via corresponding heatmaps. Finally, a comprehensive analysis was conducted to assess the relationship between immune cell infiltration and hub genes associated with atherosclerosis.

### GSVA enrichment analysis

The assessment of 50 HALLMARK pathways between the control group and patients diagnosed with atherosclerosis was performed using the “GSVA” package in the R software. GSVA analysis method was utilized for this purpose. To utilize the GSVA package, the gene set “h.all.v7.0.symbols” was extracted from the MSigDB database, which is accessible at http://software.broadinstitute.org/gsea/msigdb/index.jsp [21]. Subsequently, Spearman correlation analysis was conducted to determine the associations between hub genes, infiltrating immune cells, and the aforementioned 50 HALLMARK pathways.

### Construction of diagnosis prediction models

Following the identification of overlapping key module genes associated with unstable atherosclerotic plaques and mitochondria-related genes, a subsequent machine learning-based filtering approach was applied to refine the selection of hub genes. Subsequently, the diagnostic efficacy of these refined hub genes was assessed using receiver operating characteristic (ROC) curve analysis.

### Clinical sample collection and assessment of plaque stability

Carotid atherosclerotic plaques were obtained from patients undergoing carotid endarterectomy. Samples were collected intraoperatively and immediately divided into two components: atherosclerotic plaque and macroscopically intact arterial tissue. Each component was further subdivided, with one portion promptly frozen in liquid nitrogen (LN2) for RNA analysis, and the other portion utilized for histological examination. This study enrolled a total of 72 subjects, including 24 healthy controls, 24 patients with stable angina (SA), and 24 patients with ACS. SA was defined as typical exercise-induced myocardial ischemic chest pain, while ACS was defined as chest pain at rest with or without elevated cardiac troponin. All participants underwent coronary angiography. Patients with other causes of ACS (such as coronary vasospasm, coronary dissection, or non-obstructive MI, non-atherosclerotic inflammatory diseases (such as pneumonia and vasculitis), and neurological disorders associated with kynurenine 3-monooxygenase (*KMO*) (such as Parkinson’s syndrome and schizophrenia) were excluded from the study. Informed consent was obtained from all patients, and the study protocol adhered to the ethical guidelines outlined by the Ethics Committee of Guizhou Medical University Affiliated Hospital (Ethics Approval No: 2023 − 898) and the revised 1975 Helsinki Declaration(http://www.wma.net/en/30Publications/10Policies/b3/) [22]. Supplementary Table 1 contains the clinical data of the enrolled patients. Histological stability was determined using a scoring system developed by the American Heart Association (AHA) for assessing atherosclerosis, which evaluates various parameters such as bleeding, thrombosis, lipid core, fibrous tissue, chronic plaque inflammation, chronic fibrous cap inflammation, acute plaque inflammation, acute fibrous cap inflammation, foam cells, neovascularization, and cap rupture, providing an overall measure of plaque stability [23, 24]. Additional details regarding the scoring criteria can be found in Supplementary Table 2 [22].

### Animal model establishment

Male ApoE-/- mice (6 weeks old, *n* = 48) were purchased from specific pathogen free (SPF) Biotechnology (Beijing, China). The mice were randomly divided into four groups (*n* = 12 per group): (1) Negative control group (NC), (2) High-fat diet group (HF), (3) Sham group (HF + GFP-labeled neutral construct), and (4) Lentivirus KMO group (HF + Sh-KMO). The mice were housed in the SPF Animal Laboratory at Guizhou Medical University under controlled conditions with a temperature of 22–24 °C, humidity of 55–60%, and a 12-hour light/dark cycle. They had ad libitum access to food and water and were initially fed a regular diet for two weeks. Subsequently, the NC group continued on the regular diet, while the other groups were fed a HF diet comprising 16% fat and 1.3% cholesterol for the ensuing 12 weeks. At 8 weeks of age, the mice received intravenous injections of either an empty vector (GFP-labeled neutral construct) or lentivirus with KMO silencing at a dose of 2 × 10^7^ TU per mouse via the tail vein (KMO silencing lentiviral vector and the empty lentiviral vector were obtained from Shanghai Genechem Co., Ltd). Two weeks post-viral transduction, blood samples were collected from the orbital vein under sodium pentobarbital anesthesia. Some mice were euthanized via cervical dislocation to obtain the heart and aorta for assessing viral transduction efficiency. The remaining mice were euthanized at 20 weeks of age using the same method. The study strictly adhered to the recommendations of the Guide for the Care and Use of Laboratory Animals by the National Institutes of Health. The research protocol was approved by the Animal Experimental Ethics Committee of Guizhou Medical University (Protocol No. 2,402,131). All surgical procedures were performed under sodium pentobarbital anesthesia, with efforts made to minimize pain.

### Fluorescence detection after lentiviral transduction

The frozen sections were thawed at room temperature under dark conditions. After washing with phosphate-buffered saline (PBS), the sections were treated with 4’,6-diamidino-2-phenylindole (DAPI) staining solution and allowed to incubate at room temperature for 10 min under dark conditions. After washing away the DAPI staining solution with PBS, the images were examined using a fluorescence microscope (Olympus fluorescence microscope BX63).

### Lipid profile measurement

Blood samples were collected from the orbital vein of mice, and serum was assessed for low-density lipoprotein (LDL) cholesterol levels using an LDL cholesterol assay kit according to the manufacturer’s instructions.

### Cell culture

The RAW 264.7 cell line served as the experimental model and was cultured in Roswell Park Memorial Institute (RPMI) 1640 medium. The medium was supplemented with 20% fetal bovine serum (FBS) to provide essential nutrients and growth factors. Additionally, 100 U/ml of penicillin and 100 mg/ml of streptomycin were added to prevent contamination by bacteria and fungi. Cells were maintained at 37 °C in a humidified atmosphere containing 5% CO_2_. All experiments utilized cells in the logarithmic growth phase to ensure consistency and reliability for subsequent cellular assays.

### Quantitative real-time polymerase chain reaction (RT-qPCR)

Total RNA was extracted from various samples of macroscopically intact arterial tissue and carotid artery plaques using Trizol reagent (Ambion, Wilmington, USA). Subsequently, the isolated RNA was subjected to reverse transcription using a reverse transcription kit (Seville, Wuhan, China) to generate complementary DNA (cDNA). RT-qPCR was conducted using the 2×RealStar Fast SYBR qPCR Mix kit (Kangrun, Beijing, China) on an ABI Prism 7500 Sequence Detection System (Applied Biosystems, USA). Relative fold changes in mRNA expression levels of target genes across different samples were determined using the 2^−ΔΔCt^ method, with glyceraldehyde phosphate dehydrogenase (*GAPDH*) serving as the internal reference. Each sample was analyzed in triplicate to ensure accuracy and reproducibility. The primers of RT-qPCR used in this study were designed and validated by General Biotech (General Biotech, Anhui, China) (Supplementary Table 3). Statistical significance was assessed with a threshold of *P* < 0.05.

### Hematoxylin eosin (HE) and Masson’s staining

The paraffin sections, measuring 5 μm in thickness, were mounted onto glass slides and underwent dewaxing and rehydration procedures using xylene and graded alcohols, respectively. Staining of the sections was performed using the HE staining kit (Sigma-Aldrich, Darmstadt, Germany) and the Masson’s Trichrome Stain Kit (Sigma-Aldrich, Darmstadt, Germany).

### IHC staining

IHC staining commenced with the dewaxing and rehydration of the paraffin sections, followed by heat-induced antigen retrieval in 0.1 M citrate buffer solution at 94 °C for 20 min, with subsequent cooling to room temperature. Subsequently, the sections were incubated in the dark at room temperature for 25 min with 3% hydrogen peroxide to inhibit endogenous peroxidase activity. Following a 30-minute room temperature block with 3% bovine serum albumin (BSA), the sections were incubated overnight at 4 °C with primary antibodies, including *KMO* antibody (Proteintech, Illinois, USA), 5’-nucleotidase domain containing 3 (*NT5DC3*) antibody (Bioss, Beijing, China), acyl-CoA dehydrogenase long chain (*ACADL*) antibody (Proteintech, Illinois, USA), solute carrier family 25 member 4 (*SLC25A4*) antibody (Affinity, Wuhan, China), aldehyde dehydrogenase 1 family member B1 (*ALDH1B1*) antibody (Proteintech, Illinois, USA), monoamine oxidase B (*MAOB*) antibody (Proteintech, Illinois, USA), and *GAPDH* antibody (Affinity, Wuhan, China). After washing, the sections were exposed to the secondary antibody, goat anti-rabbit IgG-HRP (Solarbio, Beijing, China), at room temperature for 90 min. Following additional washing steps, the sections were stained with 3,3’-diaminobenzidine and counterstained with hematoxylin. Images of the stained sections were captured using the Olympus fluorescence microscope (BX63), and subsequent image analysis was performed using ImageJ software (2.14.0/1.54f).

### Immunofluorescence staining

Carotid atherosclerotic plaques from human subjects were fixed in a 4% paraformaldehyde solution and then embedded in optimal cutting temperature compound. Using a Leica cryostat, 8-µm sections were prepared from the embedded tissues. After air-drying for 1 h at room temperature, the sections were hydrated with 1×PBS for 5 min. Tissue slide preparation involved applying sodium citrate antigen retrieval buffer (PR30001, Proteintech, Wuhan, China), followed by three sequential PBS washes. Slides were then blocked with a solution containing 1% BSA and 0.25% Triton X100 in PBS at room temperature for 1 h [25]. RAW264.7 cells were cultured in six-well plates. After removing the medium, the cells were washed thrice with phosphate-buffered saline with tween (PBST). They were then fixed with 4% paraformaldehyde for 15 min, permeabilized with 0.5% Triton X-100 for 20 min, and subsequently blocked with the blocking solution at room temperature for 1 h. Following the blocking step, the samples were incubated overnight at 4 °C with primary antibodies: anti-KMO (Proteintech; catalog number: 10698-1-AP; diluted 1:1000), CD68 (Proteintech; catalog number: 25747-1-AP; diluted 1:1000-1:8000), and TOM20 (Abcam; catalog number: ab56783; diluted 1:500–1:1000). After washing, the samples were incubated with fluorescence-labeled secondary antibodies for 30 min. Subsequently, the samples were stained with DAPI dye for 5 min and washed three times with PBS. Images were captured using an Olympus fluorescence microscope (BX63), and recorded using ImageJ software (2.14.0/1.54f).

### Western blotting

Tissue lysates were prepared by extracting total protein using ristocetin-induced platelet aggregation (RIPA) lysis buffer supplemented with 1% (v/v) protease inhibitor cocktail. The lysates were then denatured by boiling in sodium dodecyl sulfate (SDS) loading buffer. Equal volumes of the denatured samples were resolved on 10% or 12% sodium dodecyl sulfate-polyacrylamide gel electrophoresis (SDS-PAGE). Subsequently, the separated proteins were transferred onto polyvinylidene fluoride (PVDF) membranes (Millipore, Billerica, MA, USA). After blocking with 5% skim milk at room temperature for 1 h, the membranes were washed with Tris-buffered saline-tween (TBST) for 30 min. Next, the membranes were incubated overnight at 4 °C with primary antibodies. Following another round of washing with TBST for 30 min, the membranes were incubated with secondary antibodies at room temperature for 1 h. Finally, the protein bands were visualized using an enhanced chemiluminescence (ECL) system and quantified using ImageJ software (2.14.0/1.54f).

### ELISA

Currently, high-sensitivity C-reactive protein (hs-CRP) has been the most commonly used serological marker for identifying characteristics of unstable plaques [26–28]. In this study, serum levels of KMO and hs-CRP were assessed using ELISA in normal controls, patients with SA, and those with ACS. According to the manufacturer’s instructions, the levels of *KMO* and hs-CRP in human serum samples were measured using an ELISA kit.

### Statistical analyses

Statistical analysis was performed using SPSS (Version 27.0). Data were presented as mean ± standard deviation. The normality of data distribution was assessed with the Shapiro-Wilk test. Normally distributed data were analyzed using a t-test; Otherwise, the Kruskal-Wallis test was applied. Group comparisons were conducted using one-way analysis of variance (ANOVA). The diagnostic performance of hub gene expression levels was evaluated using ROC curve analysis. Bioinformatics and ROC curve analyses were conducted with R software (version 4.3.0). A *P*-value < 0.05 was considered statistically significant.

## Results

### Data preprocessing

The gene expression profiles from the GSE28829, GSE41571, GSE43292, and GSE111782 datasets underwent initial normalization procedures, including standardization of data formats, imputation of missing values, and removal of outliers. Subsequently, the merged dataset was generated by combining these datasets and addressing any interbatch discrepancies. The resulting combined expression matrix comprised 11,240 gene symbols from 90 atherosclerotic plaque samples and 32 macroscopically intact arterial tissue samples within the training set. To focus on genes with significant expression variance, we selected the top 25% of genes for subsequent analysis using WGCNA. Further details and results are available in Supplementary Table 4. Additionally, Supplementary Table 5 provides disease grouping information for the 122 samples.

### Weighted gene co-expression networks analysis

**Fig. 2:**
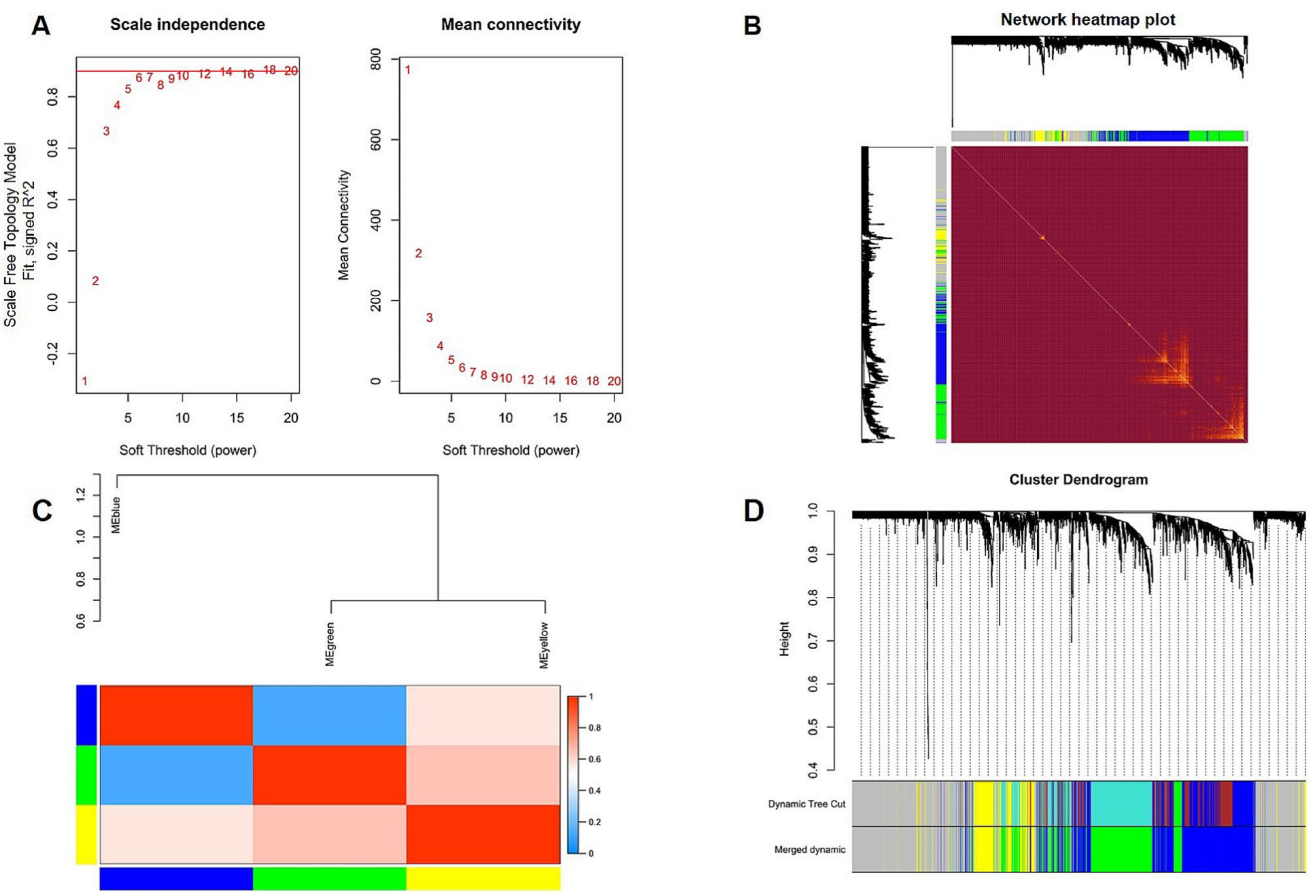
Weighted gene co-expression network analysis. (**A**) Analysis of network topology for various soft-thresholding powers. (**B**) Relationship among all modules. (**C**) Heatmap of the topological overlap in the gene network. (**D**) Clustering dendrogram of genes. Gene clustering tree (dendrogram) obtained by hierarchical clustering of adjacency-based dissimilarity

Our analysis revealed a strong correlation among genes, with the correlation coefficient exceeding 0.9, indicating their suitability for constructing multiple gene modules (Fig. 2A) using a soft threshold of β = 14. To capture the similarity between genes based on their expression profiles, we constructed a TOM by calculating correlation and adjacency matrices. The resulting gene cluster tree (Fig. 2B) visually represents the hierarchical relationships and clustering patterns among the genes. Using a hierarchical average linkage clustering method in conjunction with TOM, we successfully identified distinct gene modules within each gene network. Figure 2C depicts this process in the heatmap. Furthermore, utilizing the dynamic tree cut algorithm, we further partitioned the gene modules into three sub-modules (Fig. 2D). These findings were biologically relevant for studying gene regulatory networks and functional modules.

### Identification of relevant gene modules

**Fig. 3:**
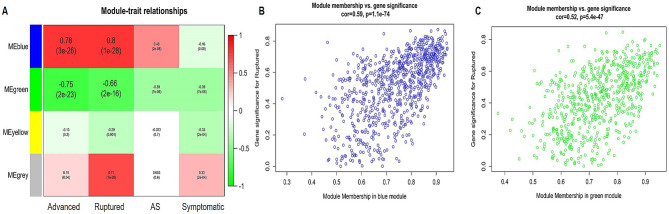
Association of module features and relationships between gene significance and module membership. Panel (**A**) represents module eigengenes arranged in rows, while clinical phenotypes are organized in columns. The correlation coefficient is presented in the first line of each cell, accompanied by the corresponding p-value displayed in the second line. The table is color-coded according to the correlation strength, as indicated in the colour legend. Panels (**B**) and (**C**) display scatterplots demonstrating a highly significant correlation between gene significance (GS) and module membership (MM) in the blue and green modules, respectively, in relation to unstable atherosclerotic plaque

Gene modules closely associated with clinical features often carry significant and specific biological implications. In Fig. 3A, the blue and green modules demonstrated robust correlations with plaque rupture, progression, and atherosclerosis. The blue module exhibited positive correlations with atherosclerotic plaque rupture (*r* = 0.80, *P* = 1E-28) and progression (*r* = 0.78, *P* = 3E-26), while the green module displayed significant negative correlations with plaque rupture (*r* = -0.66, *P* = 2E-16) and progression (*r* = -0.75, *P* = 2E-23) in the context of atherosclerosis. Further analyses underscored the strong correlation between the blue module and GS (*r* = 0.59, *P* = 1.1E-74) (Fig. 3B), as well as the correlation between the green module and GS (*r* = 0.52, *P* = 5.4E-47) (Fig. 3C). Supplementary Table 6 provides comprehensive details, including gene symbols, GS values, and corresponding *P*-values within the blue and green modules.

### Machine learning-based identification of hub genes

**Fig. 4:**
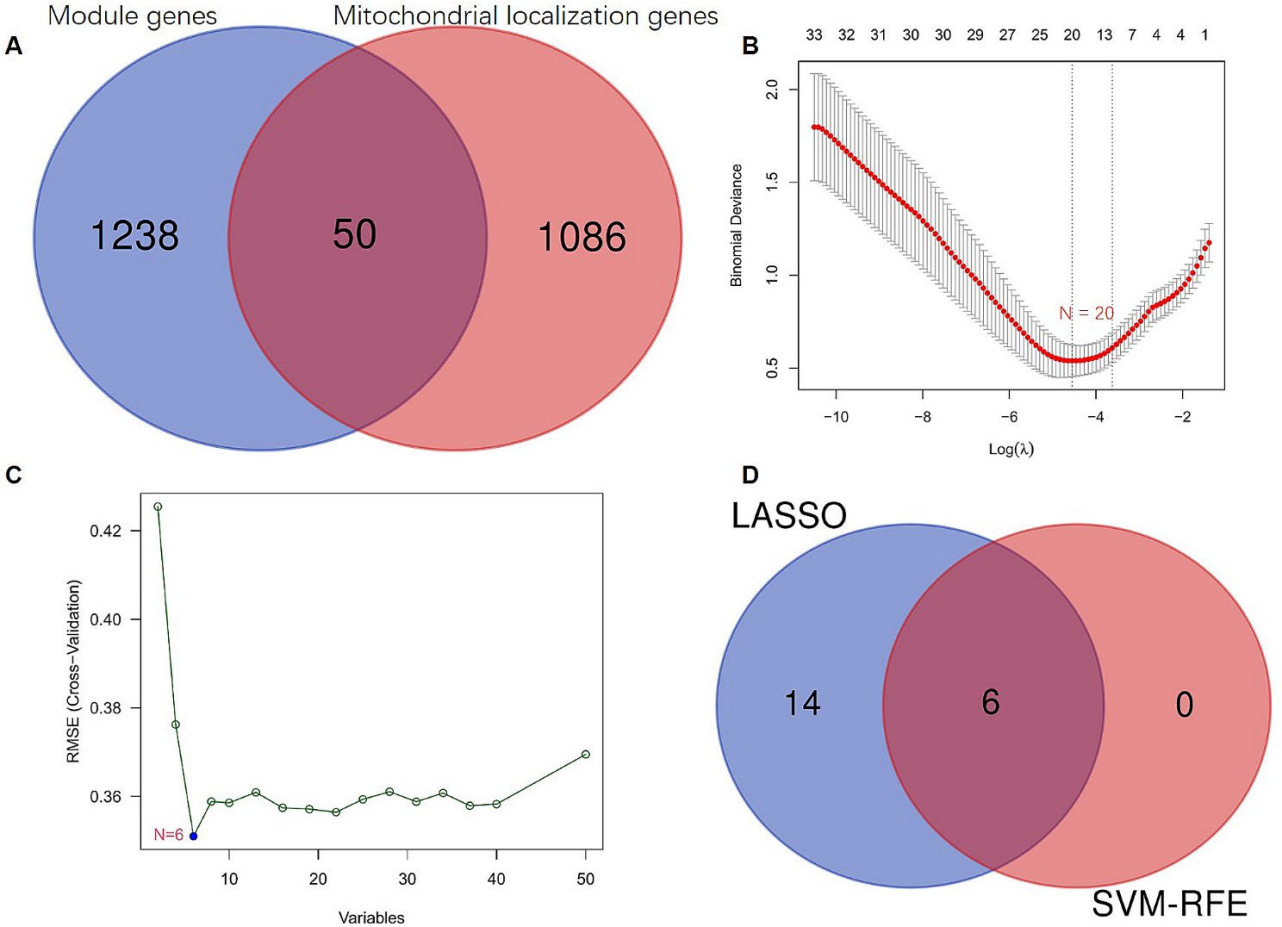
Identification of key genes in ruptured atherosclerotic plaque by machine learning. (**A**) The Venn diagram illustrates the overlapping expression of 50 genes between module genes and genes associated with mitochondrial localization. (**B**) A total of 20 key genes associated with unstable atherosclerotic plaque were identified using LASSO regression. (**C**) The SVM-RFE algorithm identified a total of 6 key genes associated with unstable atherosclerotic plaque. (**D**) The intersection of LASSO and SVM-RFE methods revealed a set of 6 key genes associated with ruptured atherosclerotic plaque. LASSO, least absolute shrinkage and selection operator; SVM-RFE, support vector machine-Recursive feature elimination

Initially, a Venn intersection analysis was conducted to identify the overlap between 1,288 genes significantly associated with unstable atherosclerotic plaques and a set of 1,136 mitochondrial genes (Supplementary Table 7). This analysis yielded a subset of 50 mitochondrial genes (as illustrated in Fig. 4A and documented in Supplementary Table 8). Subsequently, based on LASSO regression, we refined this set and identified 20 key genes from the 50 mitochondrial genes (Fig. 4B). Furthermore, using the SVM-RFE algorithm, an additional 6 key genes were identified (Fig. 4C). Finally, by intersecting the results obtained from these two machine learning approaches, 6 hub mitochondrial genes were uncovered: *NT5DC3*, *ACADL*, *SLC25A4*, *ALDH1B1*, *MAOB*, and *KMO* (Fig. 4D and Supplementary Table 9).

### Characterization of immune cell subtype distribution pattern

**Fig. 5:**
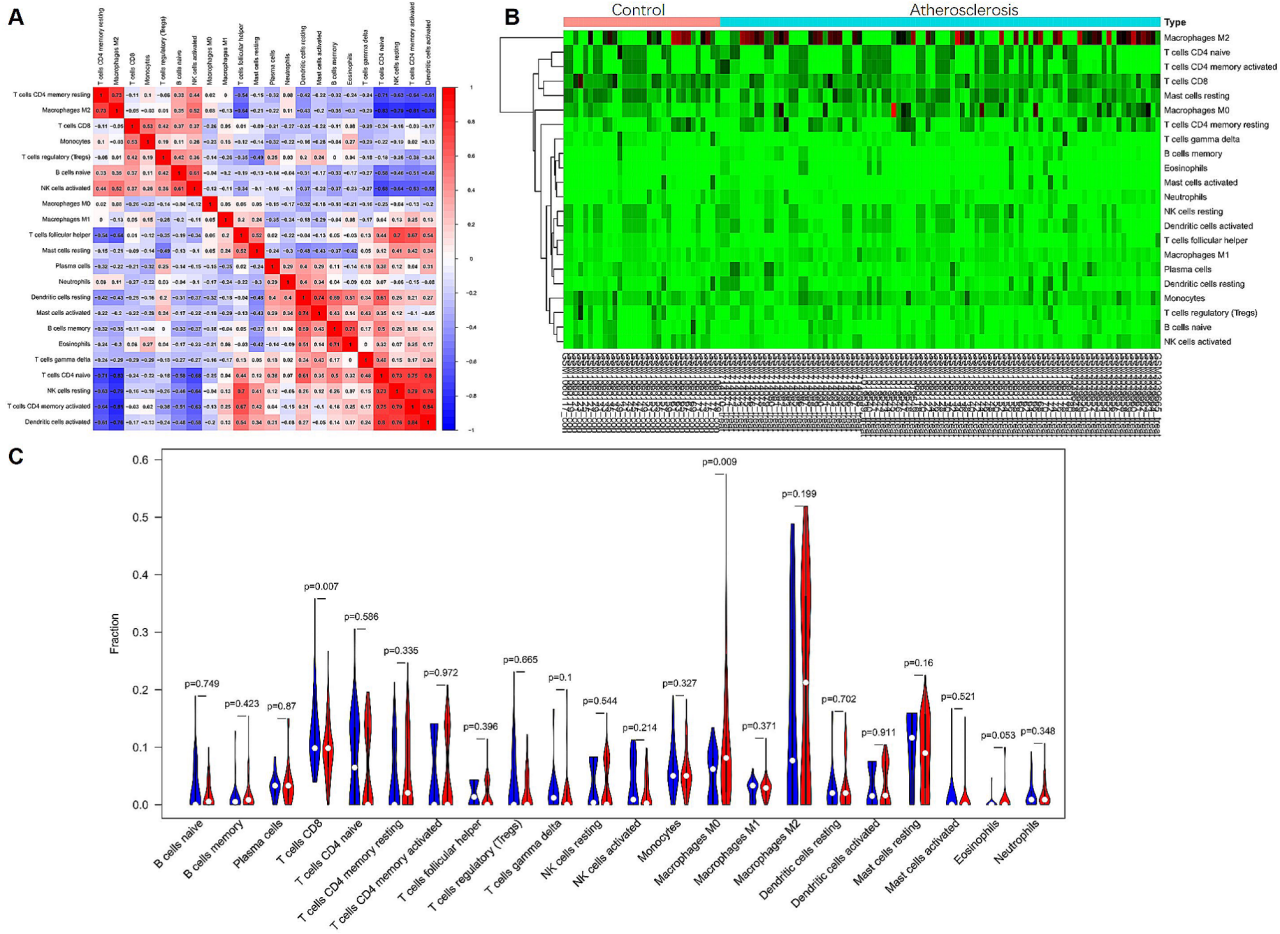
Analysis of immune cell subtype infiltration patterns between control and atherosclerosis samples. Panel (**A**) shows a correlation heatmap displaying the interrelationships among all 22 immune cell types. Panel (**B**) presents a heatmap illustrating the proportions of 22 immune cell types between control and atherosclerosis samples. Panel (**C**) presents a violin plot depicting the differential fractions of infiltrated immune cells across the 22 types between control and atherosclerosis samples

**Fig. 6:**
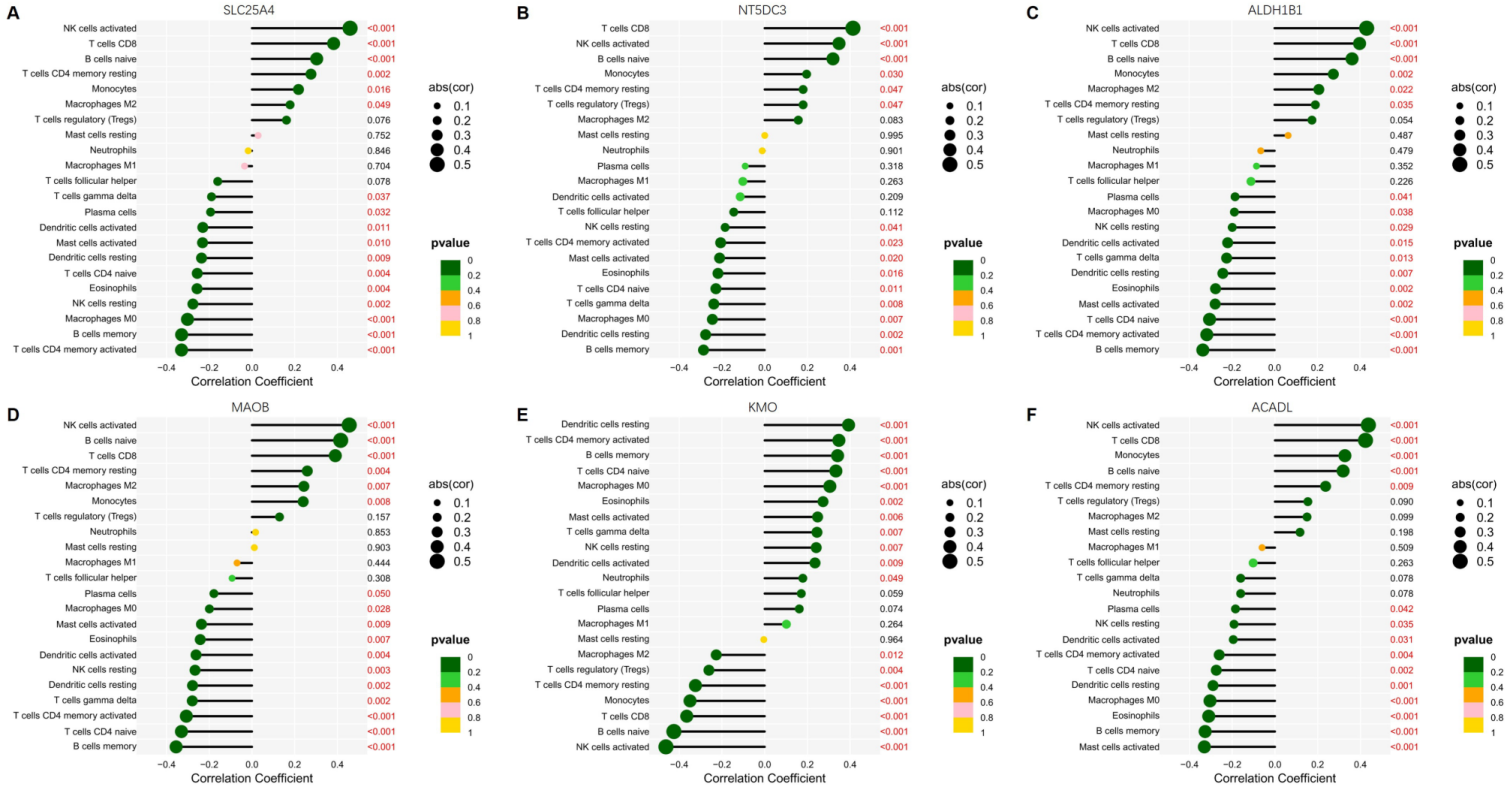
Pearson correlation analysis. Correlation between *SLC25A4* (**A**), *NT5DC3* (**B**), *ALDH1B1* (**C**), *MAOB* (**D**), *KMO* (**E**), and *ACADL* (**F**) and infiltrating immune cells was assessed. Dot size indicates the strength of correlation between genes and immune cells, with larger dots indicating stronger correlations. Dot colour represents the corresponding p-value, with greener colours indicating lower p-values. A significance threshold of *P* < 0.05 was considered statistically significant

The immune cell subtype distribution pattern was evaluated using the CIBERSORT algorithm to compare the differential expression between the control and atherosclerosis samples. Analysis of the correlation matrix revealed a negative correlation between CD8 T cells and M0 macrophages (Fig. 5A). The heatmap analysis (Fig. 5B) demonstrated significant variations in the proportions of immune cells between the control and atherosclerosis samples. The atherosclerosis samples exhibited a general decrease in CD8 T cell infiltration and an increase in M0 macrophage infiltration (Fig. 5C) (*P* < 0.01). Supplementary Table 10 provides detailed information on the immune cell infiltration pattern observed in the atherosclerosis patient group. Further analysis (Fig. 6) revealed that *SLC25A4*,* NT5DC3*,* ACADL*,* ALDH1B1*, and *MAOB* were positively correlated with CD8 T cells (*P* < 0.001) and negatively correlated with M0 macrophages (*P* < 0.001). Conversely, *KMO* displayed a negative correlation with CD8 T cells (*P* < 0.001) and a positive correlation with M0 macrophages (*P* < 0.001).

### Comparative analysis of 50 HALLMARKS pathways

Differential analysis was conducted to examine the distribution of immune cell subtypes in patients with atherosclerosis. The differences in HALLMARKS pathways between the control group and atherosclerosis patients were compared using GSVA (Supplementary Fig. 1A). Additionally, the correlation between 6 hub genes and 50 HALLMARKS pathways was evaluated (Supplementary Fig. 1B). Compared to the control group, the atherosclerosis patient group exhibited significantly increased enrichment levels in multiple pathways (*P* < 0.001). These pathways included upregulation of KRAS signaling, inflammatory response, transplant rejection, Interleukin 2 (*IL-2*)*/* Signal Transducer and Activator of Transcription 5 (*STAT5*) signaling, coagulation, heme metabolism, downregulation of ultraviolet (UV) response, *P53* signaling, reactive oxygen species, Phosphoinositide 3-kinase (*PI3K*)*/* Protein kinase B (*Akt*)*/* mammalian target of Rapamycin (*mTOR*) signaling, complement, interferon-alpha response, interferon-gamma response, myogenesis, apoptosis, Tumor Necrosis Factor alpha (*TNFA*) signaling via nuclear factor-kappaB (*NF-KB*), hypoxia, Interleukin 3 (*IL-3*)*/* Janus kinase (*JAK*)*/* Signal Transducer and Activator of Transcription 3 (*STAT3*) signaling, and xenobiotic metabolism pathways. Furthermore, *SLC25A4*,* NT5DC3*,* ACADL*,* ALDH1B1*, and *MAOB* were found to be negatively correlated with multiple pathways, such as xenobiotic metabolism, upregulation of UV response, downregulation of UV response, unfolded protein response, *TNFA* signaling via *NFKB*, *TGF-β* signaling, spermatogenesis, reactive oxygen species, *PI3K/Akt/mTOR* signaling, peroxisomes, pancreatic beta cells, *P53* signaling, myogenesis, *MYC* targets V2 signaling, *mTORC1* signaling, upregulation of *KRAS* signaling, downregulation of *KRAS* signaling, interferon-alpha response, interferon-gamma response, inflammatory response, *IL-2/STAT5* signaling, *IL-3/JAK/STAT3* signaling, hypoxia, heme metabolism, hedgehog signaling pathway, glycolysis, estrogen response late, DNA repair, complement, coagulation, cholesterol homeostasis, apoptosis, apical surface, adipogenesis, transplant rejection, angiogenesis, and androgen response. In contrast, *KMO* exhibited a positive correlation with all of the aforementioned pathways (Supplementary Table 11).

### *KMO* as a potential biomarker for assessing the instability of atherosclerotic plaques

The predictive diagnostic performance was assessed through ROC curve analysis for machine learning-based selection of central gene expression levels. As depicted in Supplementary Fig. 2, the respective AUC values for *SLC25A4*, *NT5DC3*, *ALDH1B1*, *MAOB*, *KMO*, and *ACADL* were as follows: 0.843 (with a 95% confidence interval [CI] of 0.747–0.919), 0.845 (with a 95% CI of 0.759–0.924), 0.809 (with a 95% CI of 0.701–0.899), 0.8 (with a 95% CI of 0.703–0.888), 0.823 (with a 95% CI of 0.728–0.906), and 0.84 (with a 95% CI of 0.74–0.918), respectively.

To further validate the diagnostic value of these biomarkers in assessing the instability of atherosclerotic plaques, we conducted a study involving the collection of human carotid atherosclerotic plaque samples. Plaque stability was assessed using a semiquantitative scoring system for histological grading based on the AHA classification (Supplementary Table 2). HE staining revealed different characteristics in the stable plaque group (SAP) in contrast with the control group, including an increased presence of foam cells, smaller lipid cores, and decreased infiltration of inflammatory cells. Conversely, the unstable plaque group (UAP) exhibited larger atherosclerotic plaques, tissue rupture, neovascularization, and heightened inflammatory cell infiltration.

**Fig. 7:**
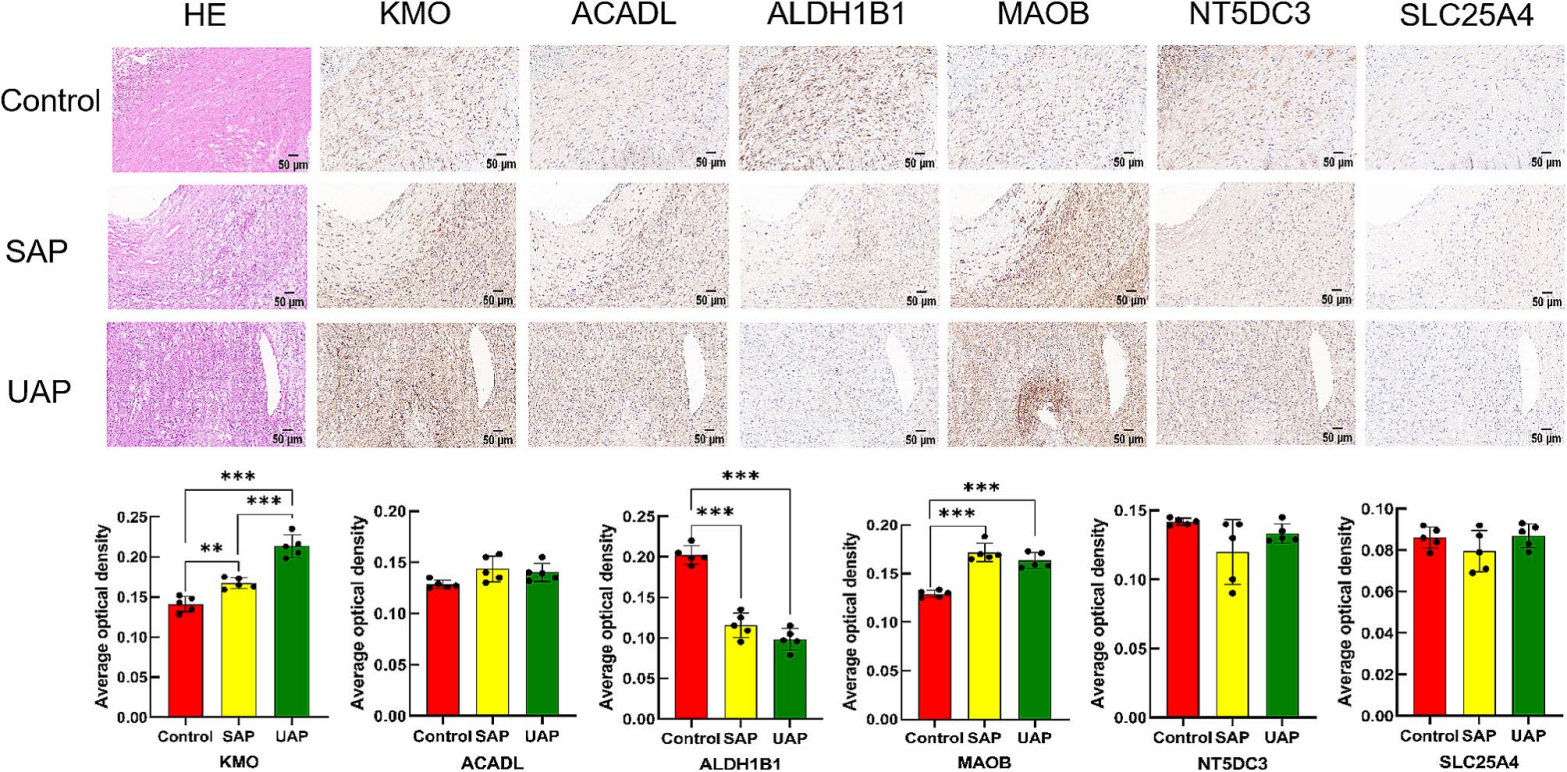
Immunohistochemistry staining was performed to preliminarily evaluate the expression of *KMO* (**A**), *ACADL* (**B**), *ALDH1B1* (**C**), *MAOB* (**D**), *NT5DC3* (**E**), and *SLC25A4* (**F**) between samples from SAP, UAP and control groups. Statistical analysis was conducted on data from the control, SAP, and UAP groups, with a sample size (n) of 5 for each group. Significance levels were indicated as ^*^ for *P* < 0.05, ^**^ for *P* < 0.01, and ^***^ for *P* < 0.001. Scale bars represent 50 μm. HE, Hematoxylin and Eosin; SAP, stable plaques; UAP, unstable plaques; Control, macroscopically intact arterial tissues

IHC was performed to preliminarily evaluate the expression of *KMO*, *ACADL*, *ALDH1B1*, *MAOB*, *NT5DC3*, and *SLC25A4* in human carotid plaque tissues (Fig. 7). Our findings revealed that *KMO* expression varied among group comparisons of the control, SAP, and UAP groups, with significant differences not only between control group and SAP group (*P* < 0.01) but also between the control group and the UAP group (*P* < 0.001). Moreover, when comparing the SAP group with the UAP group, *KMO* expression exhibited significant discrepancies (*P* < 0.001). Similarly, *MAOB* and *ALDH1B1* expressions in the control group also showed significant differences from both SAP and UAP groups (all *P* < 0.001), but no significant distinctions were observed between the SAP group and the UAP group (*P* > 0.05).

**Fig. 8:**
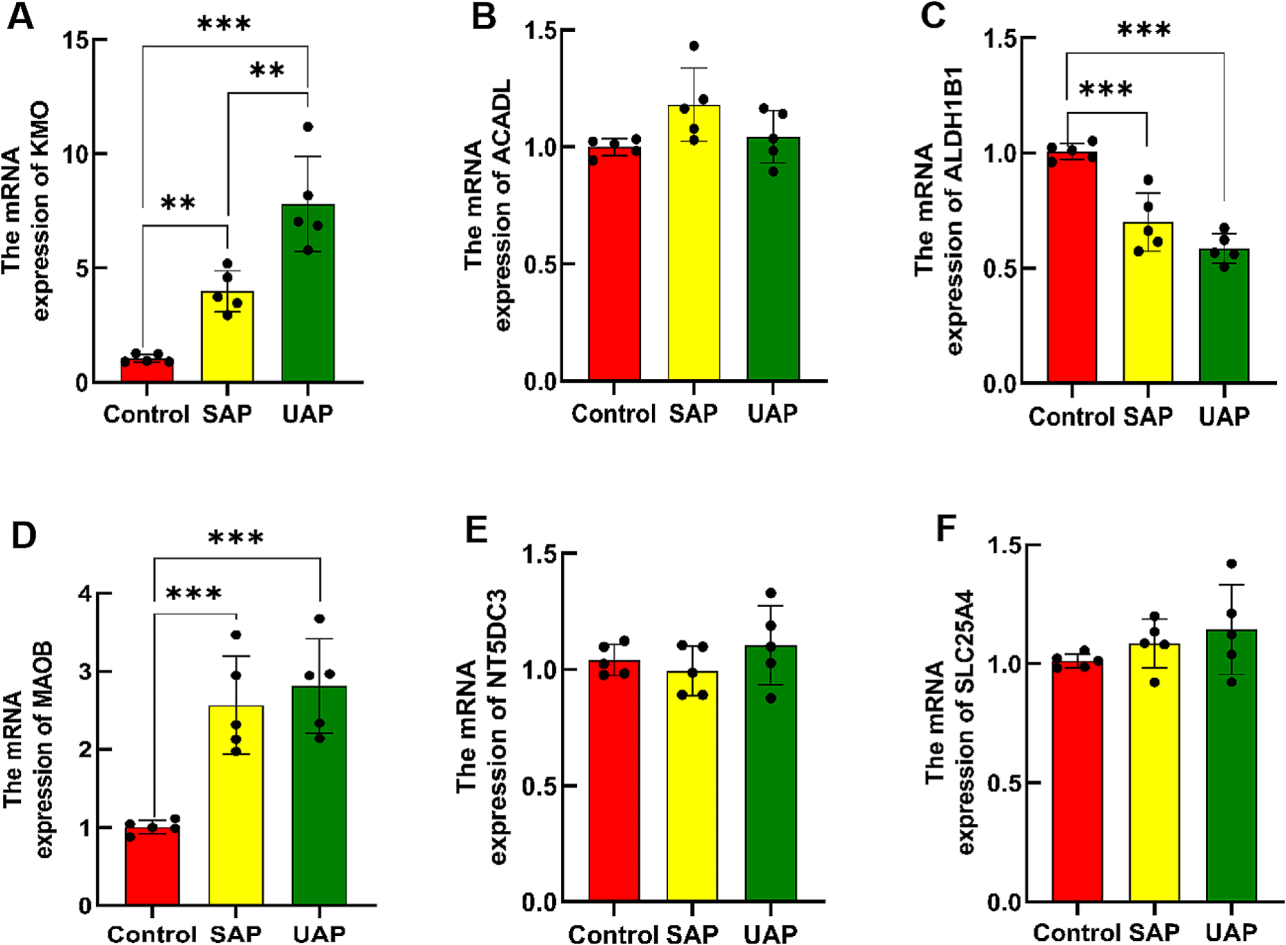
RT-qPCR analysis was performed to measure the relative RNA expression levels of *KMO*, *ACADL*, *ALDH1B1*, *MAOB*, *NT5DC3*, and *SLC25A4* between samples from SAP, UAP, and control groups. Significance levels were indicated as ^*^ for *P* < 0.05, ^**^ for *P* < 0.01, and ^***^ for *P* < 0.001

**Fig. 9:**
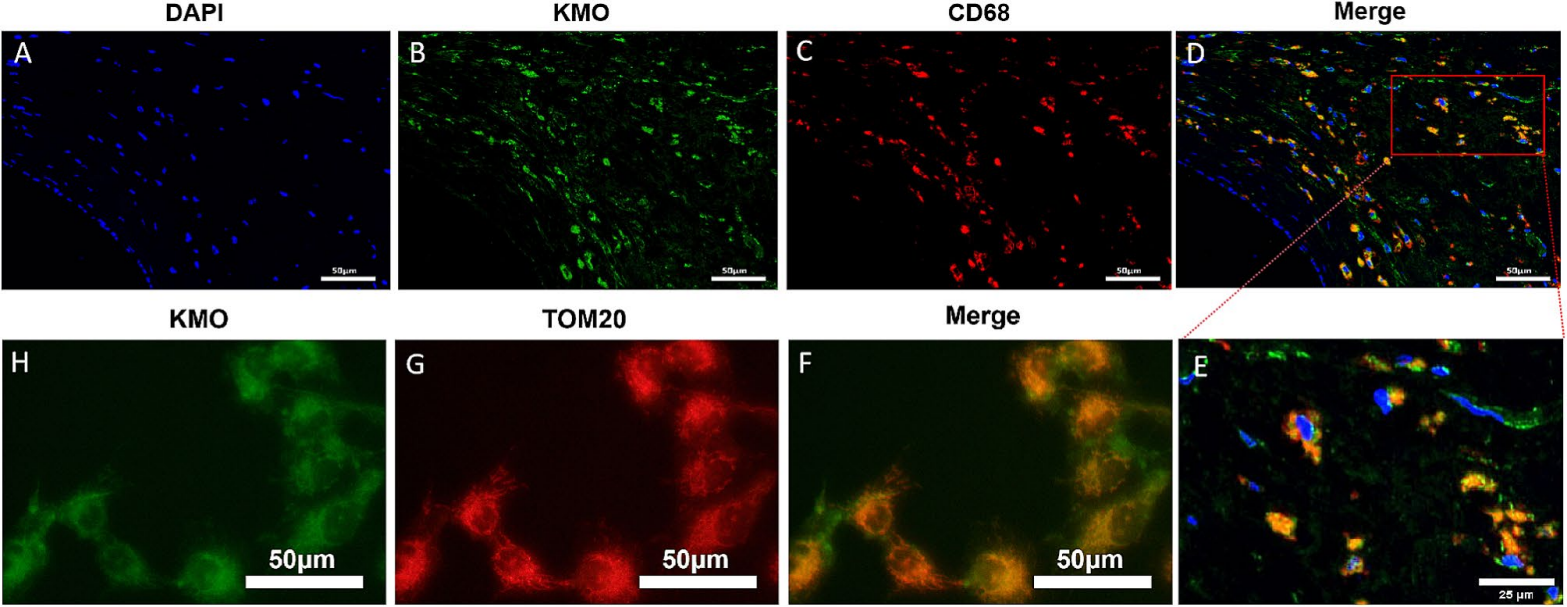
*KMO* demonstrated dual localization, with co-localization observed in macrophages and mitochondria. Immunofluorescence staining of *CD68* and *KMO* in unstable plaques of human carotid arteries was visualized. Representative images from three samples each of atheroma were analyzed. Scale bars represent 50 μm and 25 μm (magnification), respectively (**A-E**). Co-localization analysis of *KMO* and the mitochondrial-specific antibody *TOM20* in the RAW264.7 cell line demonstrated overlapping signals within macrophage mitochondria (**F-H**). Representative images from three samples each of macrophages were analyzed. Scale bars represent 50 μm

**Fig. 10:**
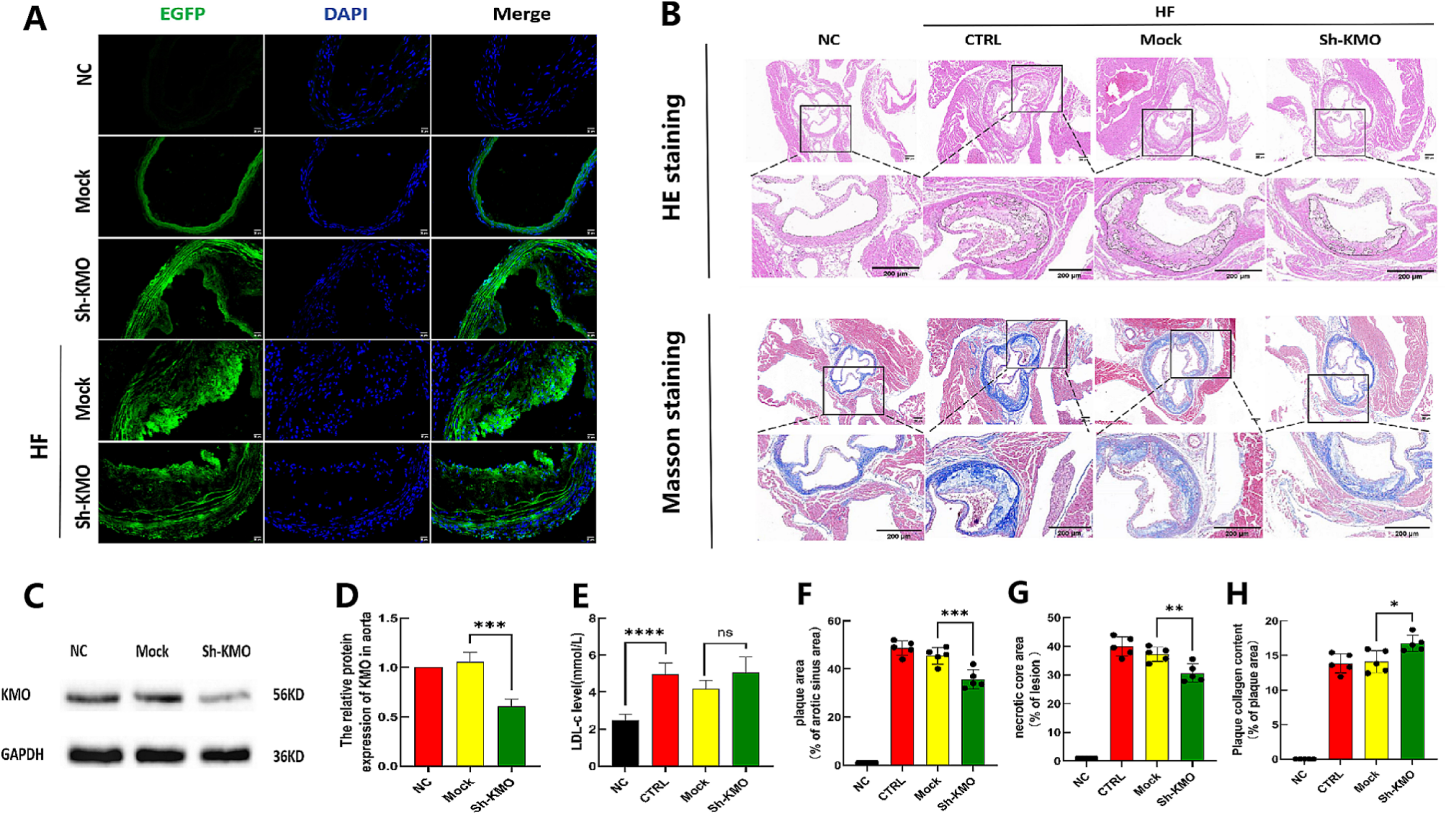
Silencing lentiviruses targeting *KMO* were successfully transfected into the aortas of ApoE-/- mice, resulting in reduced plaque formation and enhanced plaque stability in high cholesterol diet (HCD)-fed ApoE-/- mice. (**A**) Successful transfection of *KMO* silencing lentiviruses into the aortas of ApoE-/- mice (scale bar: 20 μm). (**B**) Representative images of HE and Masson’s trichrome staining in the aortic root (scale bar: 200 μm). Black boxes indicate the magnified regions below (scale bar: 200 μm). (**C**) Representative Western blot analysis of *KMO* in the aortas. (**D**) Quantification analysis of *KMO* protein levels in the aortas. (**E**) Low-density lipoprotein cholesterol (LDL-C) levels. (**F**) Quantification analysis of plaque area in the aortic root lesions using HE staining. (**G**) Quantification analysis of necrotic core area in the aortic root lesions using HE staining. (**H**) Quantification analysis of collagen content in the aortic root lesions using Masson’s trichrome staining. Data are presented as means ± standard error of the mean (SEM). *n* = 5, ^*^ for *P* < 0.05, ^**^ for *P* < 0.01, ^***^ for *P* < 0.001, and ^****^ for *P* < 0.0001. NC: mice fed a regular chow diet; CTRL: mice fed a high-fat diet; Mock: mice transfected with empty lentiviral vector + HF diet; Sh-*KMO*: mice transfected with *KMO* silencing lentiviruses + HF diet; HE: hematoxylin and eosin staining; LDL-C: low-density lipoprotein cholesterol

Concurrently, the RT-qPCR results showed that *KMO* mRNA expression level in the control group was significantly different from that in both the UAP group (*P* < 0.01) and the SAP group (*P* < 0.01); besides, significant differences were also observed between the SAP group and the UAP group (*P* < 0.001) (Fig. 8A and 9). Similarly, *ALDH1B1* and *MAOB* mRNA expressions in the control group were significantly different from those in both the SAP group (*P* < 0.001) and the UAP group (*P* < 0.001), while no significant differences were observed between the SAP group and the UAP group (*P* > 0.05) (Fig. 10C, D).

Both IHC and RT-qPCR results consistently indicated a significant upregulation of *KMO* expression in atherosclerotic stable and unstable plaques compared to the control group, with an obvious distinction between the SAP group and UAP group. Similarly, *MAOB* expression exhibited a significant upregulation compared to the control group. However, no significant differences were observed between the SAP group and UAP group. Conversely, *ALDH1B1* expression demonstrated a significant decrease in both stable and unstable plaques of atherosclerosis, with no significant differences between the SAP group and UAP group. Therefore, the *KMO* gene might hold promise as a potential diagnostic and predictive biomarker for identifying unstable plaques.

### *KMO*: dual localization in macrophages and mitochondria

To explore the subcellular localization of *KMO* in human atherosclerotic plaque tissue and cells, we conducted immunofluorescence co-staining of *KMO* and the macrophage marker *CD68* in unstable plaques of human carotid arteries (Fig. 9A-E). The results revealed the co-localization of *KMO* within *CD68*-positive macrophages, consistent with the findings derived from our study utilizing WGCNA, machine learning, and immune cell infiltration analysis on a publicly available dataset of human carotid atherosclerosis. To further validate *KMO* as a mitochondrial gene, we cultured the RAW264.7 cell line and performed co-staining of *KMO* with the mitochondrial-specific antibody *TOM20*. Co-localization analysis of *KMO* and the mitochondrial-specific antibody *TOM20* demonstrated their overlapping signals within macrophage mitochondria (Fig. 9F-H). These findings provided compelling evidence for *KMO* expression in macrophages within atherosclerotic lesions and the role of *KMO* as a mitochondrial gene.

### Attenuation of atherosclerotic plaque formation and enhancement of plaque stabilization through silencing of *KMO* in HCD-fed ApoE^−/−^ mice

In ApoE^−/−^ mice, we detected the expression of the construct in the aorta following lentiviral transduction at both 2 and 20 weeks. Frozen sections of the aortic root exhibited EGFP fluorescence (Fig. 10A). Western blot analysis revealed a significant difference in the aortic *KMO* protein levels between the groups (Fig. 10C and D), indicating successful Sh-KMO transduction in the aortic wall of ApoE^−/−^ mice. After 20 weeks of a HF diet, the ApoE^−/−^ mice were euthanized, and blood samples were collected from the orbital vein. Histological analysis of the aortic root sections using HE and Masson’s Trichrome staining demonstrated that the HF diet significantly increased the aortic plaque area (Fig. 10B top and F), necrotic core area (Fig. 10B top and G), collagen-positive area (Fig. 10B bottom and H), and serum LDL cholesterol levels (Fig. 10E) in ApoE^−/−^ mice. The HE staining revealed that the Sh-KMO transduced mice had reduced aortic plaque area (Fig. 10A and F) and necrotic core area (Fig. 10B top and G) compared to the control group. Masson’s Trichrome staining showed that the Sh-KMO transduced mice had increased collagen-positive area (Fig. 10B bottom and H), suggesting that *KMO* promotes the formation and instability of atherosclerotic plaques.

### *KMO* as a diagnostic biomarker for ACS

**Fig. 11:**
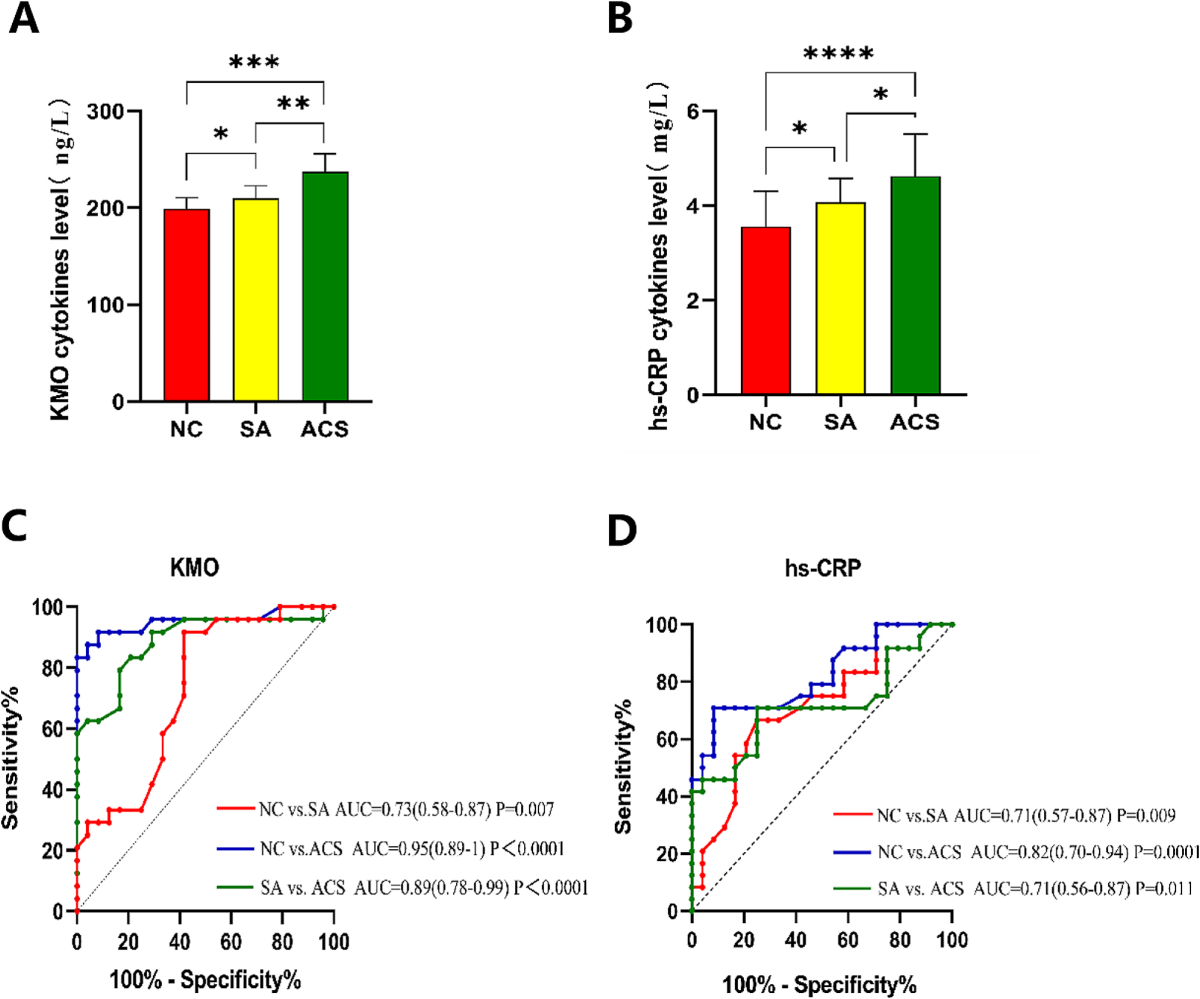
Results of ELISA analysis. (**A**) Serum *KMO* levels in control samples and patients with SA or ACS. (**B**) Serum hs-CRP levels in control samples and patients with SA and ACS. (**C**) ROC curve analysis based on serum *KMO* levels. (**D**) ROC curve analysis based on serum hs-CRP levels. *KMO*, kynurenine 3-monooxygenase; SA, stable angina; ACS, acute coronary syndrome; ROC, Receiver operating characteristic; hs-CRP, high-sensitivity C-reactive protein

Compared to the control group, patients with SA exhibited significant elevations in serum *KMO* and hs-CRP levels. Similarly, patients with ACS had significantly higher levels of both *KMO* and hs-CRP compared to the control group, with even greater differences observed when comparing ACS to SA (Fig. 11A-B). The mean *KMO* levels were 198.8 ng/L in the control group, 210.0 ng/L in the SA group, and 237.2 ng/L in the ACS group. The mean hs-CRP levels were 3.56 mg/L in the control group, 4.07 mg/L in the SA group, and 4.63 mg/L in the ACS group. ROC curve analysis (Fig. 11C) demonstrated that the AUC for *KMO* in differentiating SA from the control group was 0.73 (95% CI 0.58–0.87, *P* = 0.007) with a sensitivity of 0.92 and a specificity of 0.58. For the comparison between ACS and the control group, the AUC for *KMO* was 0.95 (95% CI 0.89-1.00, *P* < 0.0001) with a sensitivity of 0.88 and a specificity of 0.96. When comparing ACS to SA, the AUC for *KMO* was 0.89 (95% CI 0.79–0.98, *P* < 0.0001) with a sensitivity of 0.79 and a specificity of 0.83. Additionally, the AUC for hs-CRP in differentiating SA from the control group was 0.71 (95% CI 0.57–0.87, *P* = 0.009) with a sensitivity of 0.67 and a specificity of 0.75. For the comparison between ACS and the control group, the AUC for hs-CRP was 0.82 (95% CI 0.70–0.94, *P* < 0.0001) with a sensitivity of 0.71 and a specificity of 0.87. When comparing ACS to SA, the AUC for hs-CRP was 0.71 (95% CI 0.56–0.87, *P* = 0.011) with a sensitivity of 0.71 and a specificity of 0.75 (Fig. 11D). Notably, in distinguishing between ACS and SA, the area under the AUC curve for *KMO* and its sensitivity and specificity were significantly greater than those of hs-CRP, indicating that *KMO* might have a superior ability to differentiate between ACS and SA compared to hs-CRP. These findings provided a basis for further exploration of the clinical utility of *KMO* in the diagnosis of CAD and ACS.

## Discussion

IS frequently arises from the rupture of unstable atherosclerotic plaques and subsequent thrombus formation. Identifying biomarkers to stratify the risk of stroke and therapeutic targets to stabilize vulnerable plaques in atherosclerosis is crucial for preventing adverse cerebrovascular and cardiovascular events. In this study, we obtained four datasets (GSE28829, GSE41571, GSE43292, and GSE111782) from the GEO database as training datasets and performed WGCNA. We identified two modules (blue and green) significantly associated with unstable atherosclerotic plaques. Utilizing LASSO logistic regression and SVM-RFE machine learning methods, we identified six hub genes (*NT5DC3*, *ACADL*, *KMO*, *SLC25A4*, *ALDH1B1*, and *MAOB* significantly correlated with unstable atherosclerotic plaques. ROC analysis based on the training set demonstrated the high efficacy of these six hub genes in distinguishing unstable atherosclerotic plaques. To validate our findings, we conducted IHC combined with RT-qPCR experiments. Interestingly, significant differences in *KMO* expression were observed between the control group and the atherosclerotic plaque group. Elevated *KMO* expression was associated with the promotion of atherosclerosis progression. We found a significant increase in *KMO* expression in both stable and unstable atherosclerotic plaques, with an obvious distinction between the two plaque types. These findings suggest that *KMO* may contribute to plaque instability and hold considerable value and significance in predicting plaque stability and vulnerability.

Following a thorough exploration of the NCBI Gene database, *KMO* (HGNC: 6381, gene ID: 8564, OMIM: 603,538) was identified at the chromosomal locus 1q43, characterized by 15 exons. This gene encodes a mitochondrial outer membrane protein that catalyzes the hydroxylation of L-tryptophan metabolite, L-kynurenine (KYN), yielding L-3 hydroxykynurenine (HK). Studies in yeast have recognized *KMO* as a therapeutic target for Huntington’s disease [29]. The kynurenine pathway, serving as the principal metabolic route for tryptophan, generates various metabolites with distinct biological properties [30]. This pathway plays critical roles in diverse biological processes, including immune regulation, cancer, inflammation, and metabolism [31, 32]. Within the kynurenine pathway, metabolites such as KYN, kynurenic acid (KYNA), 3-HK, and 3-hydroxyanthranilic acid (3-HAA) exert significant biological effects. *KMO*, a critical enzyme within the kynurenine pathway, facilitates the conversion of tryptophan to 3-HK and is intricately involved in immune regulation, cancer development, inflammation, and metabolism [33, 34]. Consequently, KMO is associated with various conditions, including cardiovascular diseases, neurological disorders, tumor formation, and inflammation. Overactivation of the kynurenine pathway has been observed in the presence of several cardiovascular risk factors, including obesity, hyperglycemia, dyslipidemia, hypertension, smoking, and aging [35, 36]. Furthermore, recent studies have provided compelling evidence regarding the significant involvement of KYN and its metabolites in the pathophysiology of cardiovascular diseases. Moreover, pharmacological interventions targeting the modulation of the kynurenine pathway have shown therapeutic potential in managing various cardiovascular diseases [35, 37].

Several studies have investigated the role of KMO in cardiovascular diseases. For instance, Lai et al. [38] reported a significant increase in KMO expression in myocardial cells of mice with MI, closely associated with elevated levels of xanthurenic acid. Furthermore, experimental evidence indicated that KMO influenced myocardial cell apoptosis and ferroptosis by modulating mitochondrial fission and fusion processes, thereby exacerbating MI-induced damage. In a separate study, Kubo et al. [39] demonstrated that *KMO*−/− mice exhibited enhanced survival rates and reduced inflammatory cell infiltration in a mouse model of viral myocarditis compared to *KMO* mice. Additionally, *KMO*−/− mice displayed elevated serum levels of kynurenine, anthranilic acid, and KYNA, along with decreased levels of chemokines such as chemokine ligand (CCL) 1, CCL2, CCL3, and CCL4, suggesting the potential of targeting indoleamine 2,3-dioxygenase or inhibiting *KMO* to improve myocarditis outcomes. Gellért et al. [40] found that the repeated intraperitoneal administration of *KMO* inhibitors at specific time intervals (1, 30, and 180 min) following bilateral carotid artery occlusion resulted in a significant reduction in infarct size in rats. The involvement of kynurenine and its metabolites in the pathophysiology of atherosclerosis has been extensively studied. Activation of the kynurenine pathway and elevated plasma levels of KYNA, 3-HK, anthranilic acid, and 3-HAA have been linked to an increased risk of acute myocardial infarction in patients with SA pectoris, particularly in subgroups with diabetes or prediabetes [41]. Previous research has highlighted the facilitative role of the enzymes *KMO* and kynureninase in the development of atherosclerotic plaques [41], suggesting the potential utility of measuring these metabolites and enzymes as biomarkers for identifying individuals at risk of cardiovascular complications. While no comparative research has yet investigated *KMO* expression differences specifically between stable and unstable plaques, our study revealed that *KMO* was associated not only with the formation of atherosclerotic plaques but also with their stability.

Atherosclerosis is characterized by vascular inflammation, contributing to plaque instability [42]. The groundbreaking study by Fernandez et al. provided a comprehensive morphological characterization of immune cells during atherosclerosis, elucidating the immune cell composition within plaques and delineating their distinct activation states. This seminal work laid the groundwork for investigating atherosclerosis as an immune-mediated disorder [43]. Both innate and adaptive immune cells, particularly macrophages, play significant roles in the pathogenesis of atherosclerosis. Macrophage-related pathological processes are important targets for diagnostic imaging and new therapies in atherosclerosis. Therapeutic manipulation of macrophages within atherosclerotic plaques can effectively inhibit pro-atherosclerotic macrophage activities, thereby enhancing the resolution of inflammation and stabilizing plaques [44]. T lymphocytes, a critical population of immune cells, can be classified into CD4 and CD8 subsets based on their surface markers and functions. CD8 T cells exhibit a dual role in atherosclerosis. Previous research has indicated that CD8 T cells can secrete a range of inflammatory cytokines, thereby exacerbating the inflammatory response and promoting plaque instability [45]. However, cytotoxic activity directed towards antigen-presenting cells and regulatory CD8 T cells has demonstrated efficacy in impeding the progression of atherosclerosis by mitigating immune reactions [45]. Additionally, Xu et al. [46]. documented obvious elevations in the proportions of M0 macrophages, gamma delta (γδ) T cells, and neutrophils, while observing a decrease in the proportions of eosinophils and resting dendritic cells, among patients exhibiting atherosclerosis progression leading to plaque rupture. Consistent with these findings, further exploration of immune cell population differences between atherosclerotic and normal samples revealed decreased CD8 T cell infiltration and increased M0 macrophage infiltration in atherosclerotic samples. Based on these results, it could be inferred that M0 macrophages promote the formation of atherosclerosis, while CD8 T cells may delay its progression. Previous studies have suggested that *KMO* exerts a critical regulatory role in macrophages, with emerging evidence highlighting its close interplay with macrophage inflammatory responses and immune modulation [34]. Heightened *KMO* activity augments flux within the kynurenine pathway, resulting in excessive generation of pro-inflammatory mediators, obvious quinolinic acid [47]. Consequently, this elicits robust inflammatory cascades. *KMO* activity profoundly influences macrophage functionality and inflammatory responses, thereby affecting the pathogenesis and progression of inflammatory disorders, including neuroinflammation, autoimmune diseases, and atherosclerosis [34]. Interestingly, a positive correlation between *KMO* expression and the infiltration abundance of M0 macrophages was observed. Utilizing immunofluorescence co-staining techniques, the expression patterns of *KMO* in human carotid unstable plaque tissues and cultured mouse macrophage cell lines were examined. The findings provide strong evidence that *KMO* participates in atherosclerosis by mediating macrophage function. However, additional in vivo and in vitro experiments are necessary to further elucidate the potential molecular mechanisms by which *KMO* mediates macrophage function in atherosclerosis.

Mitochondrial dysfunction is intricately associated with a spectrum of chronic human disorders, including atherosclerosis and diabetes mellitus [48]. Mitochondria, which are dynamic organelles, continuously undergo turnover within living cells. Processes of mitochondrial fission and fusion processes ensure the preservation of a functional mitochondrial population, aligned with the cellular energy demands [49]. Mitophagy, a specialized autophagic process, mediates the elimination of impaired or excessive mitochondria [50]. These complicated processes are meticulously regulated by a diverse repertoire of proteins and genes. Perturbations in any of these processes can culminate in the accumulation of dysfunctional mitochondria, compromised energy production, heightened oxidative stress, and ultimately cellular demise—consistently observed features in various human disorders [51]. Severe mitochondrial dysfunction is well-established as the causative factor in specific mitochondrial disorders, while progressive mitochondrial damage is also evident in various chronic diseases, including cancer and atherosclerosis, underscoring its prominent role in disease progression [52]. The association between mitochondrial dysfunction and atherosclerosis has garnered increasing attention in recent years. In this study, we used WGCNA to identify key genes within modules and intersected them with the mitochondrial gene set from the MitoCarta 3.0 database [18], yielding a list of 50 mitochondria-related unstable plaque genes. The identification of *KMO* as a target gene was accomplished through a combination of machine learning algorithms and experimental validation. Furthermore, we conducted cell immunofluorescence colocalization experiments to confirm the subcellular localization of *KMO* within macrophage mitochondria. In future, it is crucial to undertake additional research to explore the potential role of *KMO* in promoting atherosclerosis via mitochondrial dysfunction and elucidate the specific mechanisms underlying its regulation of mitochondrial dysfunction.

Chronic inflammation promotes the progression of plaques and increases their vulnerability, necessitating precise determination of plaque vulnerability based on specific features such as a lipid-rich core, thin fibrous cap, and intraplaque hemorrhage. Although these features can be partially identified through imaging techniques, effectively recognizing the vulnerability of arterial plaques remains challenging [53]. Therefore, investigating circulating biomarkers as supplementary indicators to predict the risk of atherosclerotic plaque rupture or vulnerability and inform subsequent revascularization is important. Previous studies have linked most biomarkers predicting plaque rupture or vulnerability primarily to inflammatory molecules, including hs-CRP, interleukin-6 (IL-6), matrix metalloproteinase 9 (MMP9), monocyte chemoattractant protein-1 (MCP-1), and CD163. Additionally, other biomarkers encompass short, single-stranded, non-protein-coding RNAs such as miR-21, miR-133, and miR-145, which may be released into circulation due to plaque instability or rupture [53]. In this context, there is increasing evidence supporting the role of inflammatory biomarkers in acute ischemic events; however, they cannot predict disease progression. Moreover, their concentrations may increase nonspecifically in other concurrent pathological conditions. Consequently, inflammation-related biomarkers for predicting plaque vulnerability exhibit higher sensitivity but lower specificity [54]. The results of this study suggest that using *KMO* as a serum biomarker demonstrates higher sensitivity and specificity in distinguishing between patients with SA and ACS compared to hs-CRP. Although our study provides some evidence indicating *KMO*’s potential as a reliable serum biomarker for predicting unstable plaques, further research is necessary to substantiate this finding. Therefore, identifying *KMO* as a reliable circulating biomarker in clinical practice for selecting patients with high-risk vulnerable plaques still requires extensive investigation.

Several limitations should be acknowledged in this study. Firstly, the inclusion of validation samples from a single center with small sample sizes may limit the generalizability of the findings. The variability in gene expression levels among individuals from diverse geographic regions or ethnic backgrounds remains unclear. Therefore, it is imperative to undertake larger sample sizes and multicenter studies to validate and enhance the robustness and generalizability of the results. Secondly, further in vivo and in vitro investigations are warranted to elucidate the underlying mechanisms governing the relationship between *KMO* and immune cell infiltration in atherosclerosis. Additionally, it is essential to explore the potential contribution of *KMO* to atherosclerosis progression through mitochondrial dysfunction and investigate the specific regulatory pathways involved. Research in these areas will provide a more comprehensive understanding of the role of *KMO* in atherosclerosis and its potential therapeutic implications.

## Conclusions

In conclusion, our study identified *KMO* as a mitochondria-targeted gene associated with the immune system, demonstrating its potential as a promising diagnostic biomarker for assessing the instability of atherosclerotic plaques. Our results indicated that M0 macrophages likely might contribute to the initiation and progression of atherosclerotic plaques, while CD8 T cells might play a protective role. Moreover, we observed a positive correlation between *KMO* expression and M0 macrophages, along with a negative correlation with CD8 T cells. These findings highlight the significant role of *KMO* in the interplay with macrophages, elucidating its relevance to the development and progression of atherosclerotic plaques. Thus, our study provides novel insights for fundamental research and unveils potential avenues for the development of preventive and therapeutic strategies targeting atherosclerosis-related diseases.

### Electronic supplementary material

Below is the link to the electronic supplementary material.

Supplementary Material 1

Supplementary Material 2

## Data Availability

The raw data supporting the conclusions of this article will be made available by the authors, without undue reservation.

## Abbreviations

WGCNA: Weighted gene co-expression network analysis
CIBERSORT: Cell-typeidentification by estimating relative subsets of RNA transcripts
GSVA: Gene SetVariation Analysis
ELISA: Enzyme-linked immunosorbent assay
GS: Gene signifi-cance
SVM-RFE: Support vector machine-recursive feature elimination
LASSO: Least absolute shrinkage and selection operator
ACS: Acute coronary syndrome
MI: Myocardial infarction
TIA: Transient ischemic attack
IS: Ischemic stroke
ApoE-/-: Apolipoprotein E knockout
GEO: Gene Expression Omnibus
AHA: American Heart Association
MM: Module membership
LN2: Liquid nitrogen
ROC: Receiver Operating Characteristic
TOM: Topological overlap matrix
SPF: Specificpathogen free
cDNA: Complementary DNA
HE: Hematoxylin and eosin
IHC: Immunohistochemistry
BSA: Bovineserumalbumin
DAB: 3,3’-diaminobenz-idine
AHA: American Heart Association
SAP: Stable plaque group
UAP: Unstable plaque group
RT-qPCR: Quantitative real time polymerase chain reaction
CD: Crohn’s disease
UC: Ulcerative colitis
AUC: Areas under the curve
NT5DC3: 5’-nucleotidase domain containing 3
ACADL: acyl-CoA dehydrogenase long chain
SLC25A4: Solute carrier family 25 member4
ALDH1B1: Aldehyde dehydrogenase 1 family member B1
MAOB: Monoamine-oxidaseB
KMO: Kynurenine3-monooxygenase
PBS: Phosphate-bufferedsaline
KYN: Kynurenin
KYNA: Kynurenicacid
3-HK: 3-hydroxykynurenine
3-HAA: 3-hydroxyanthranilic acid
XA: Xanthurenic acid
CCL: Chemokine ligand
IDO: Indoleamine2,3-dioxygenase
CKD: Chronic kidney disease
DAPI: 5 − 4’,6-4-5-diamidino-2-phenylindole
LDL: Low-density lipoprotein
RPMI: Roswell Park Memorial Institute
FBS: Fetal bovineserum
PBS: Phosphate-buffered saline
GAPDH: Glyceraldehyde phosphate dehydrog-enase
PBST: Phosphate-buffered saline with tween
RIPA: Ristocetin-induced platelet aggregation
SDS: Sodium dodecyl sulfate
SDS-PAGE: Sodium dodecyl sulfatepolyacrylamide gel electrophoresis
PVDF: Proteins were transferred onto polyvinylidene fluoride
TBST: Tris-buffered saline-tween
ECL: Enhanced chemiluminescence
hs-CRP: High- sensitivity C-reactive protein
ANOVA: One-way analysis of variance
IL-2: Interleukin 2
STAT5: Signal Transducer and Activator of Transcription 5
UV: Ultraviolet
PI3K: Phosphoinositide 3-kinase
Akt: kinase B
mTOR: Mammalian target of Rapamycin
TNFA: Tumor Necrosis Factor alpha
NF-KB: Nuclear factor-kappaB
IL-3: Interleukin 3
JAK: Janus kinase
STAT3: Signal Transducer and Activator of Transcription 3
IL-6: Interleukin-6
MMP9: Matrix metalloproteinase 9
MCP-1: Monocyte chemoattractant protein-1
EGFP: Enhanced Green Fluorescent Protein
HCD: High cholesterol diet
LDL-C: Low-density lipoprotein cholesterol
