# Supplementary material for: Identification and experimental validation of KMO as a critical immune-associated mitochondrial gene in unstable atherosclerotic plaque
Source: J Transl Med. 2024 Jul 18;22:668. doi: 10.1186/s12967-024-05464-5 (PMC11256392; doi:10.1186/s12967-024-05464-5)
Supplement: Supplementary file 1 — Supplementary Material 1 [file 12967_2024_5464_MOESM1_ESM.docx]

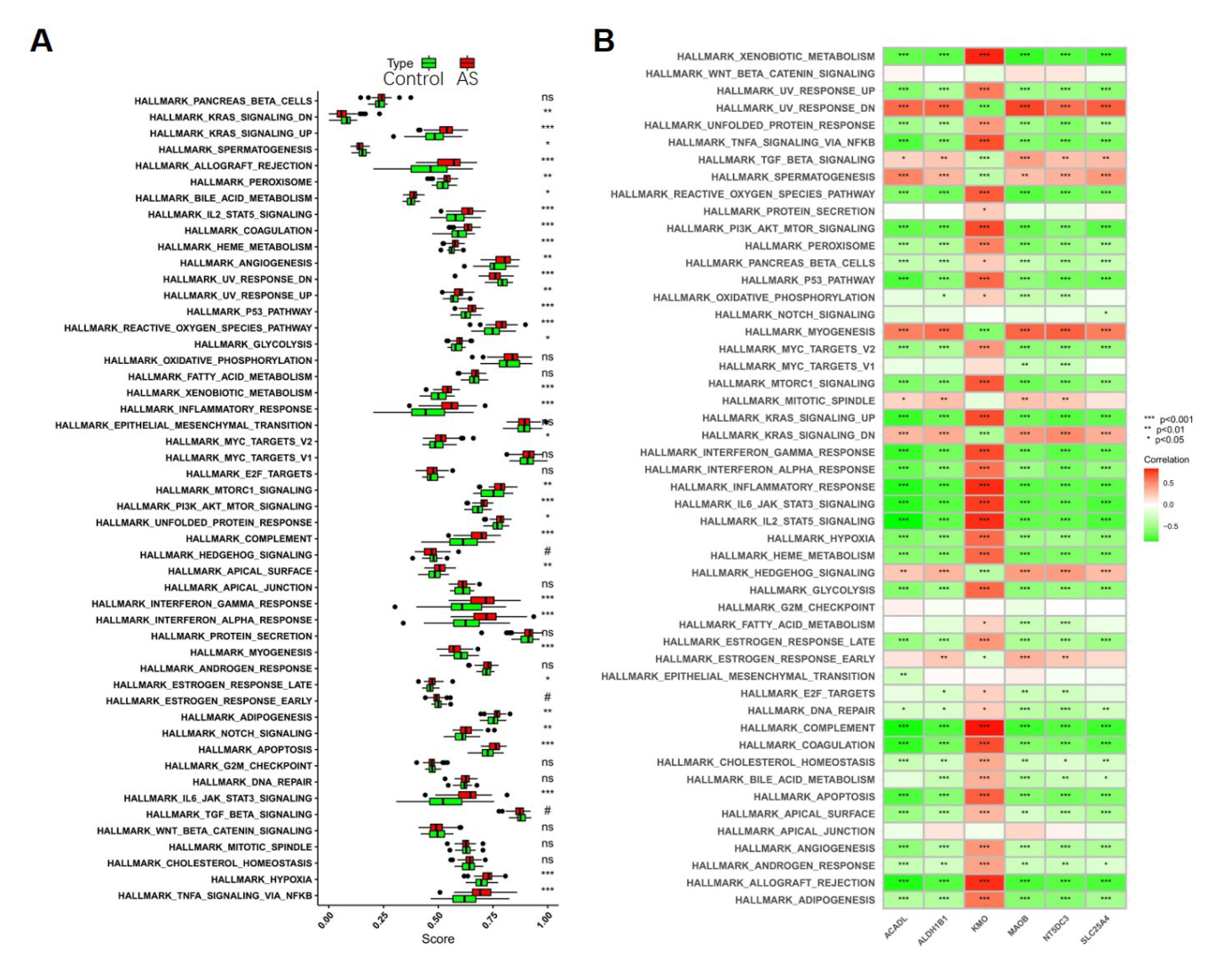
**Supplementary Figure1**. GSVA enrichment analysis.

The HALLMARKS pathways were compared between the control group and atherosclerosis patients using GSVA (**A**). Furthermore, the correlation between 6 hub genes and 50 HALLMARKS pathways was assessed (**B**). The atherosclerosis patient group demonstrated significantly elevated enrichment levels in multiple pathways compared to the control group (*P* < 0.001).


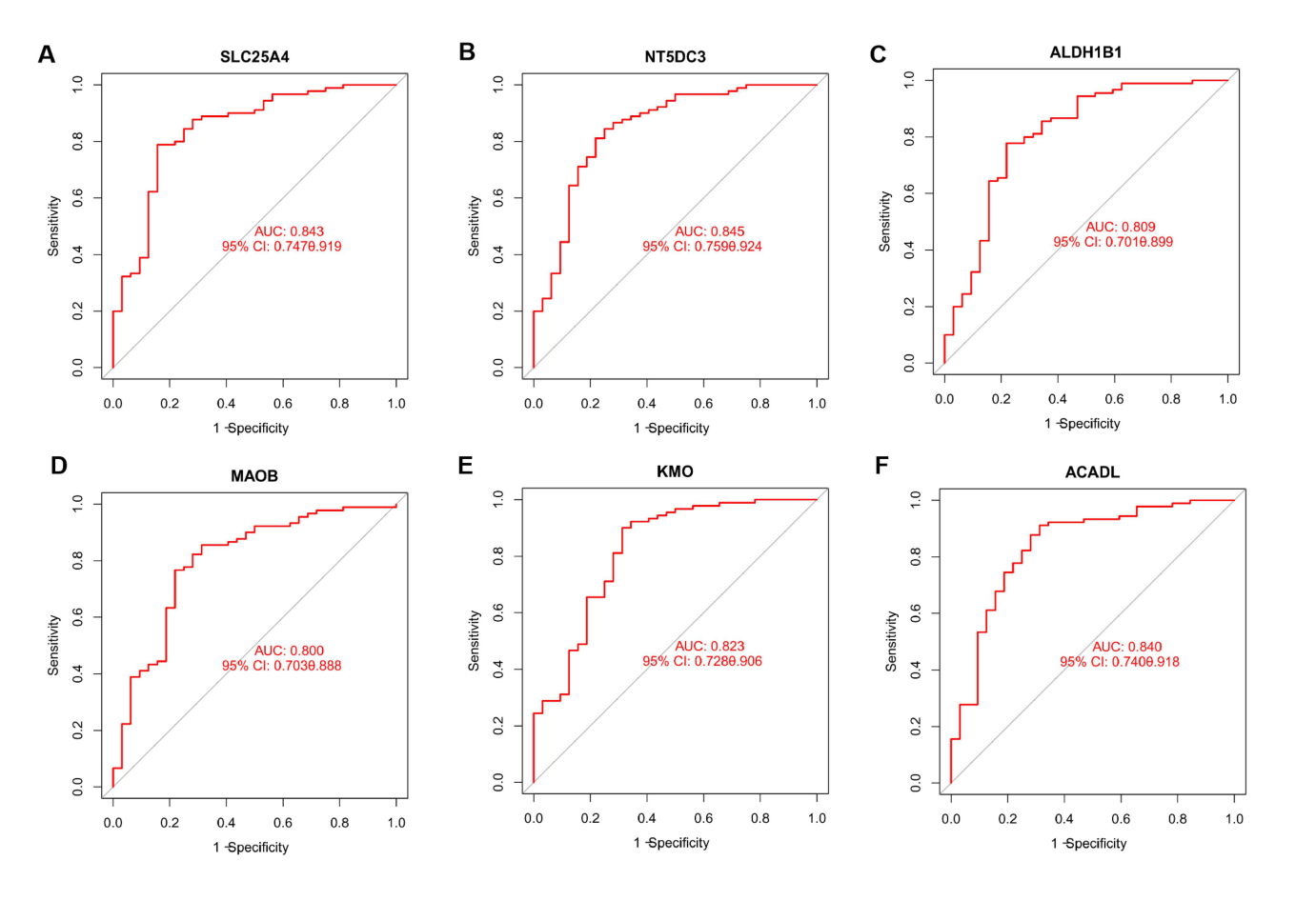


**Supplementary Figure2**. ROC curve analysis.

ROC curve analysis of *SLC25A4* (**A**), *NT5DC3* (**B**), *ALDH1B1* (**C**), *MAOB* (**D**), *KMO* (**E**), and *ACADL* (**F**) in the training set GSE28829, GSE41571, GSE43292 combined and GSE111782 (red line).
